# Supplementary material for: Neuroprotective Norsesquiterpenoids and Triterpenoids from Populus euphratica Resins
Source: Molecules. 2019 Nov 30;24(23):4379. doi: 10.3390/molecules24234379 (PMC6930471; doi:10.3390/molecules24234379)
Supplement: Supplementary file 1 [file molecules-24-04379-s001.pdf]

Supplementary Materials

Neuroprotective Norsesquiterpenoids and Triterpenoids from *Populus euphratica* Resins

Yun-Yun Liu [1,2,†], Dan-Ling Huang [1,†], Yun Dong [1], Da-Peng Qin [1], Yong-Ming Yan [1] and Yong-Xian Cheng [1,*]

1 School of Pharmaceutical Sciences, Shenzhen University Health Science Center, Shenzhen 518060, China; leonchemistry@szu.edu.cn (D.-L.H.); yundong@szu.edu.cn (Y.D.); tqindp@szu.edu.cn (D.-P.Q.); yanym@szu.edu.cn (Y.-M.Y);

2 Shanxi University of Chinese Medicine, Jinzhong 030619, China; liuyunyun2017@foxmail.com (Y.-Y.L.);

* Correspondence: yxcheng@szu.edu.cn; Tel.: +86-0755-26902073;

† These authors contributed equally to this work.

Table of Contents

1. Figures S1–S15 NMR spectra and HRESIMS of 1
2. Figures S16–S24 NMR spectra and HRESIMS of 2
3. Figures S25–S32NMR spectra and HRESIMS of 3
4. Figures S33–S40 NMR spectra and HRESIMS of 4
5. Figures S41–S43 NMR spectra of 4a
6. Figures S44–S46 NMR spectra of 4b
7. Figure S47. The lowest energy conformers of 1
8. Figure S48. The lowest energy conformers of 2
9. Figure S49. The lowest energy conformers of 3
10. Figure S50. The lowest energy conformers of 4
11. Table S1. Extracted heats and weighting factors of the optimized conformers of 1–4
12. Table S2. The Cartesian coordinates of the lowest energy conformers for 1–4

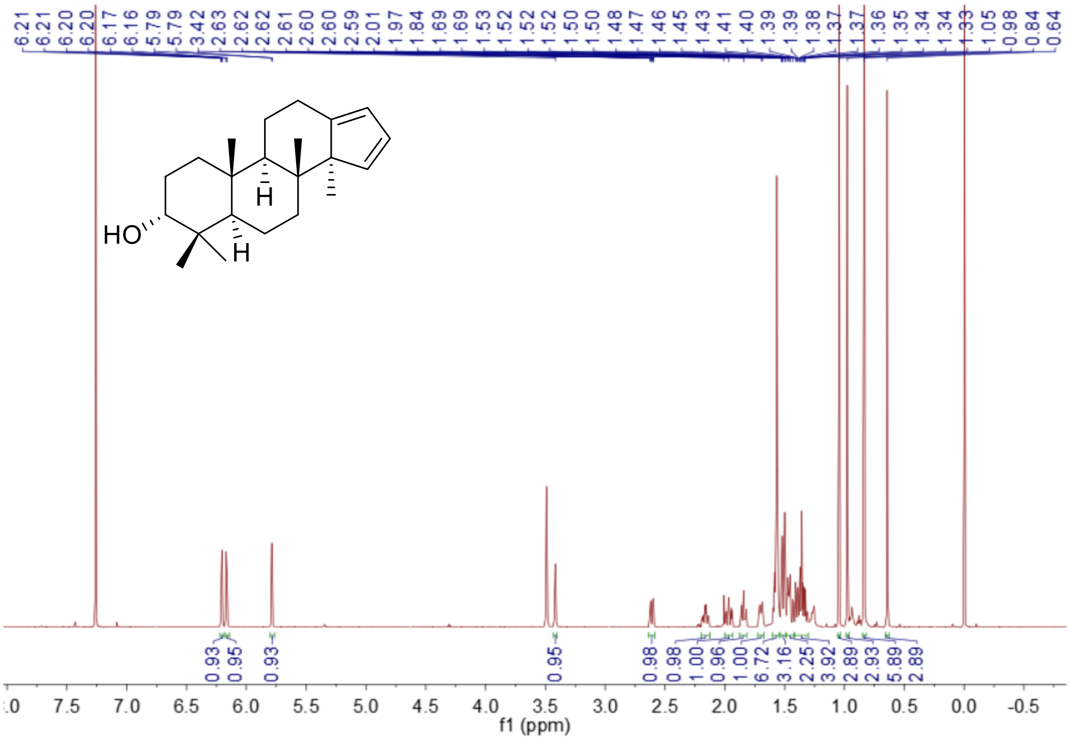

Figure S1.^{1}H NMR spectrum of **1** in $CDCl_3$.

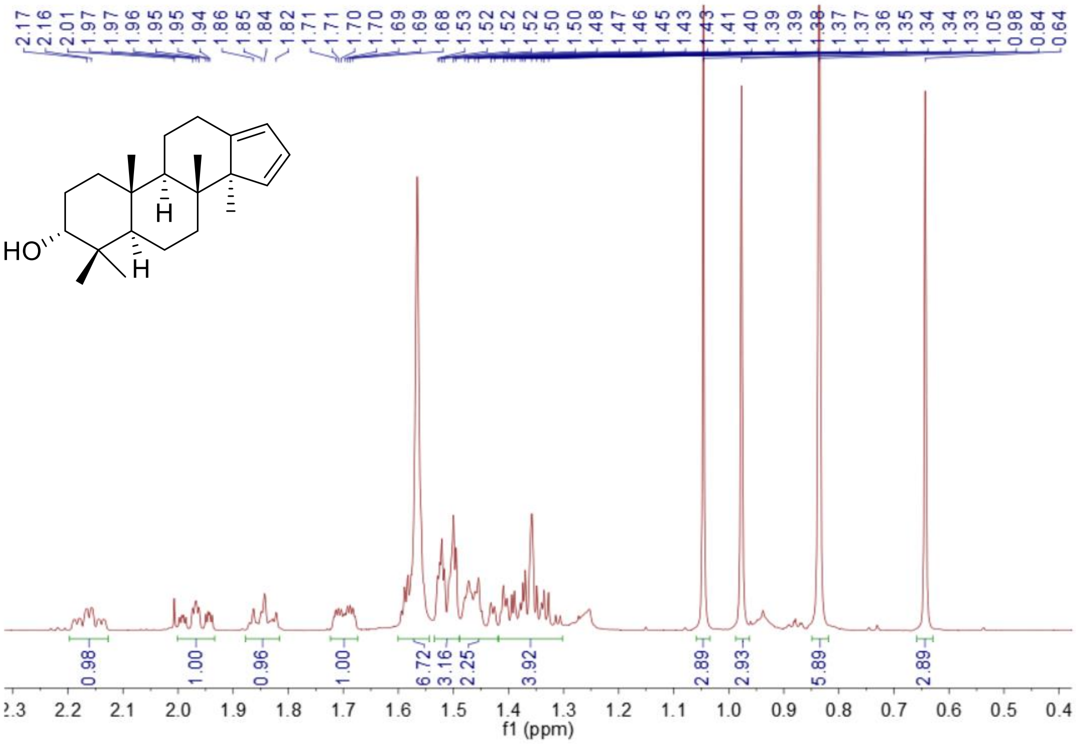

Figure S2. Enlarged ^{1}H NMR spectrum of **1** in $CDCl_3$.

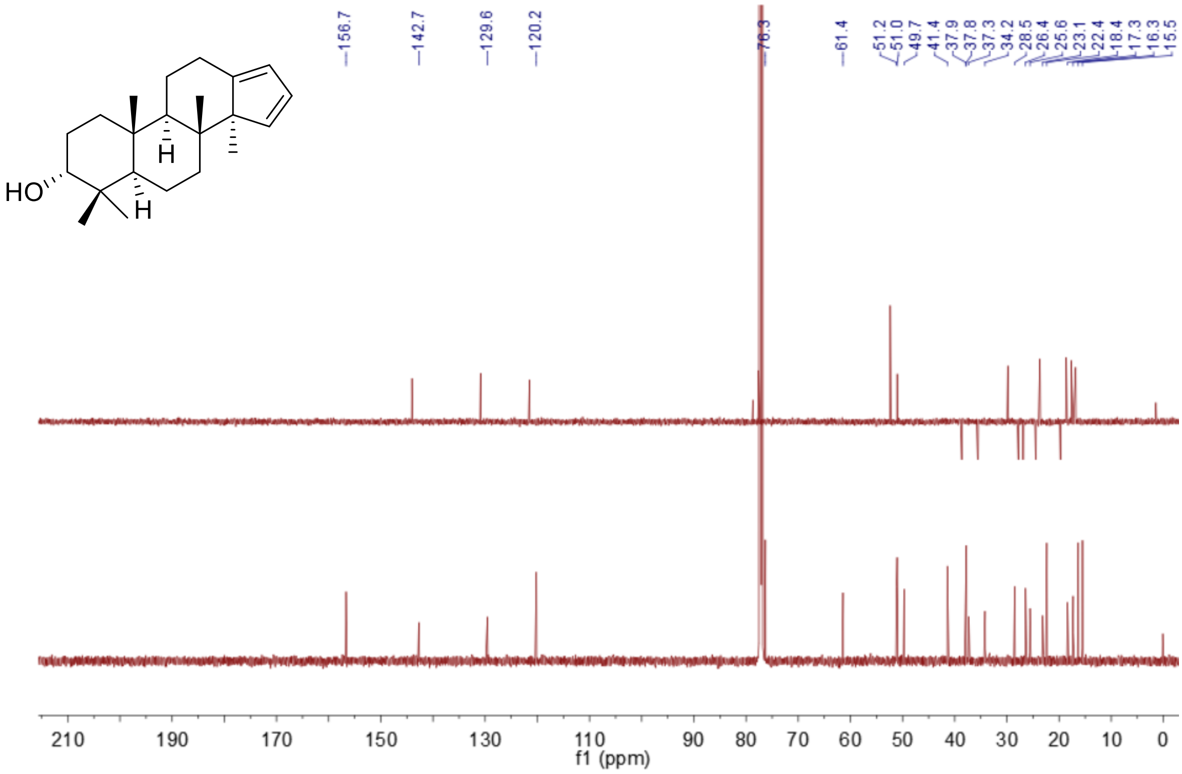

Figure S3. ^{13}C NMR and DEPT spectra of **1** in $CDCl_3$.

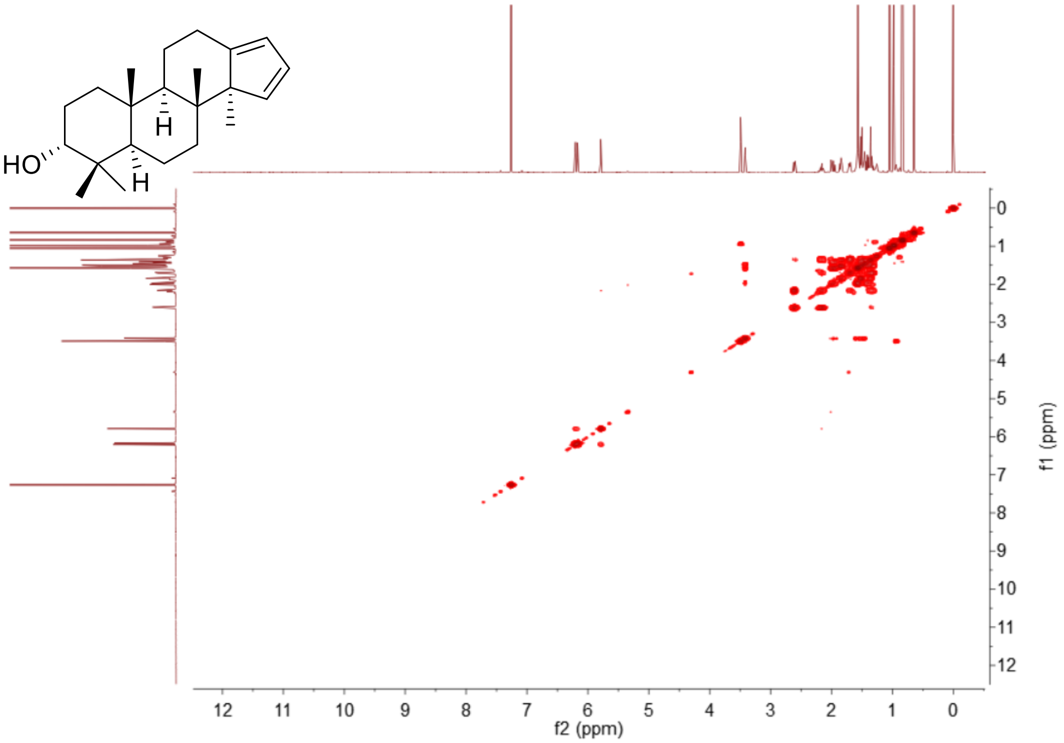

Figure S4. ^{1}H-^{1}H COSY spectrum of **1** in $CDCl_3$.

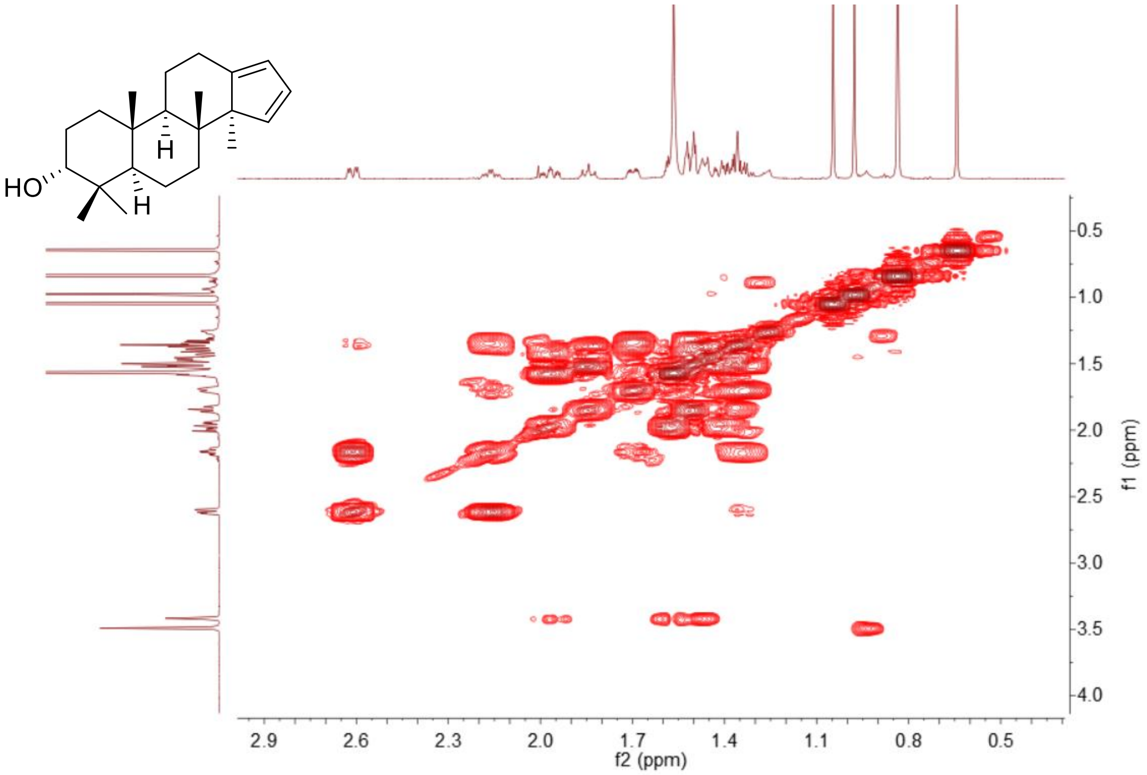

Figure S5. Enlarged ^{1}H-^{1}H COSY spectrum of **1** in $CDCl_3$.

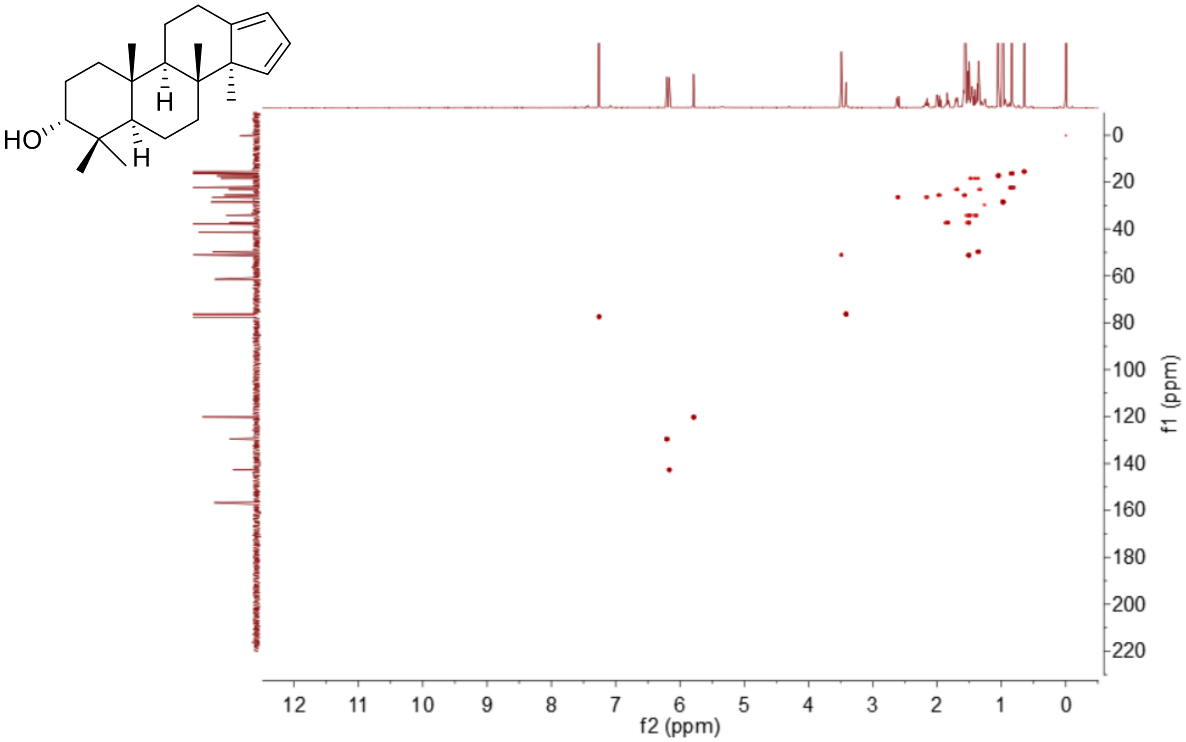

Figure S6. HSQC spectrum of **1** in $CDCl_3$.

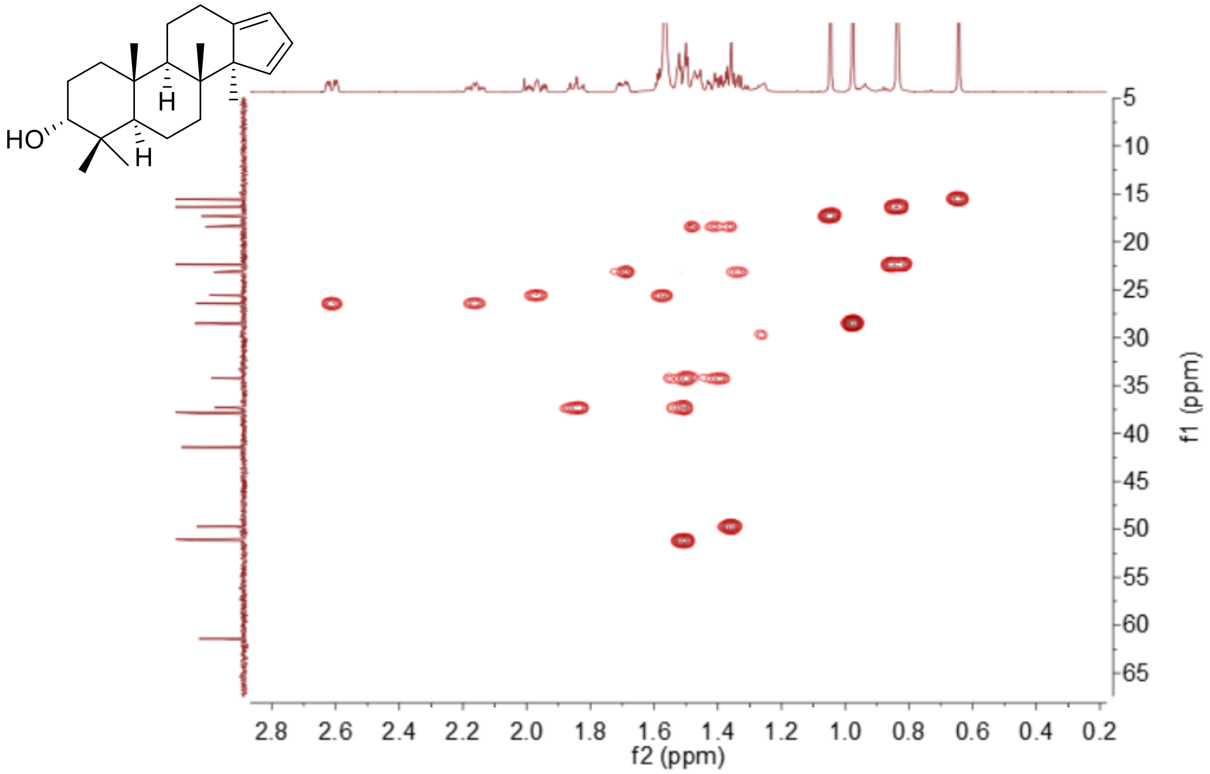

Figure S7. Enlarged HSQC spectrum of **1** in $CDCl_3$.

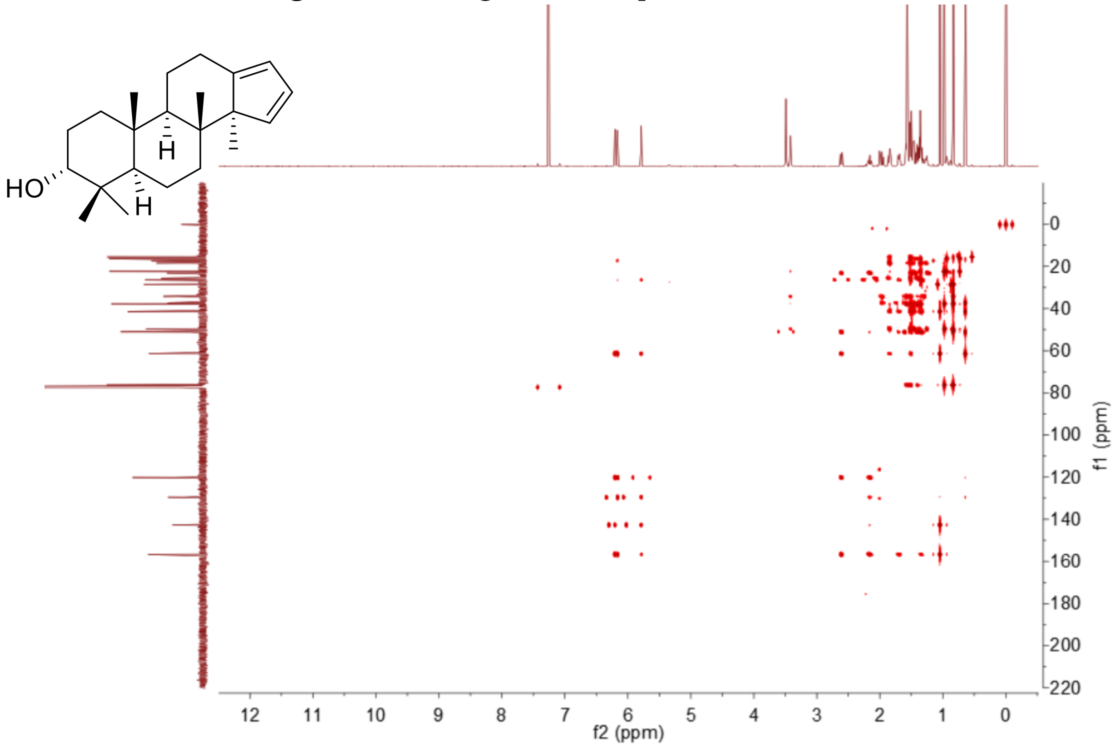

Figure S8. HMBC spectrum of **1** in $CDCl_3$.

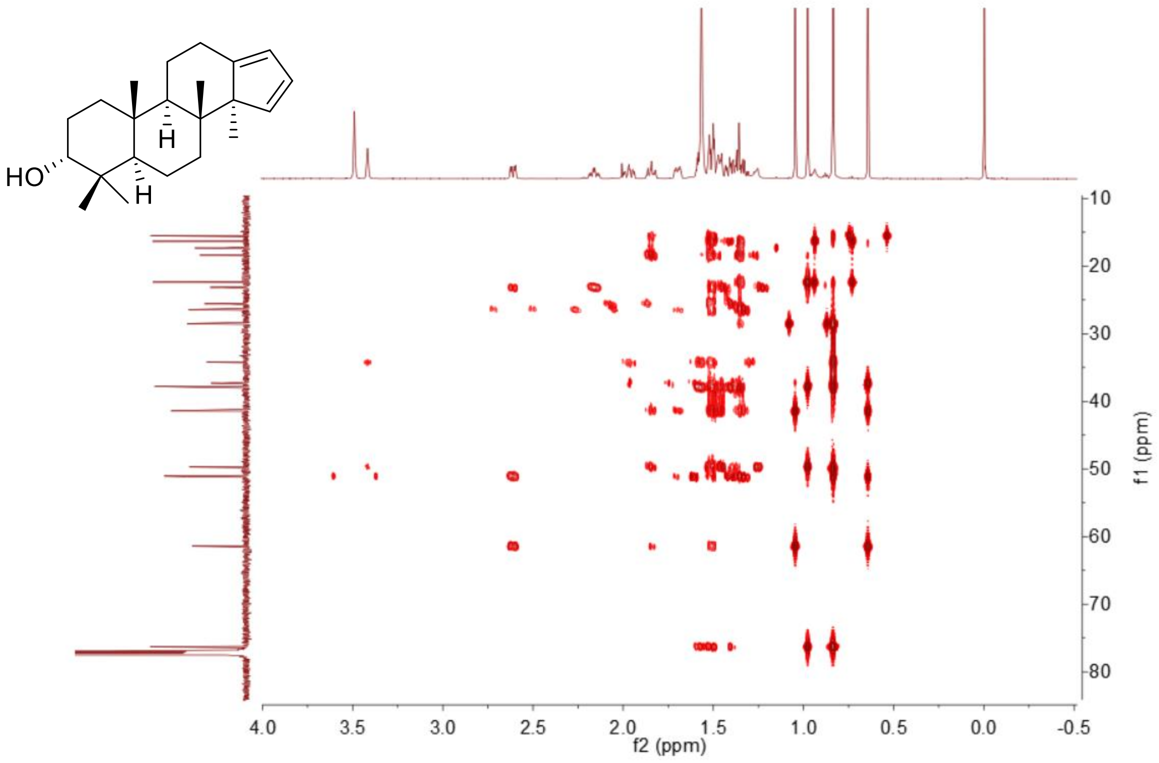

Figure S9. Enlarged HMBC spectrum of **1** in $CDCl_3$.

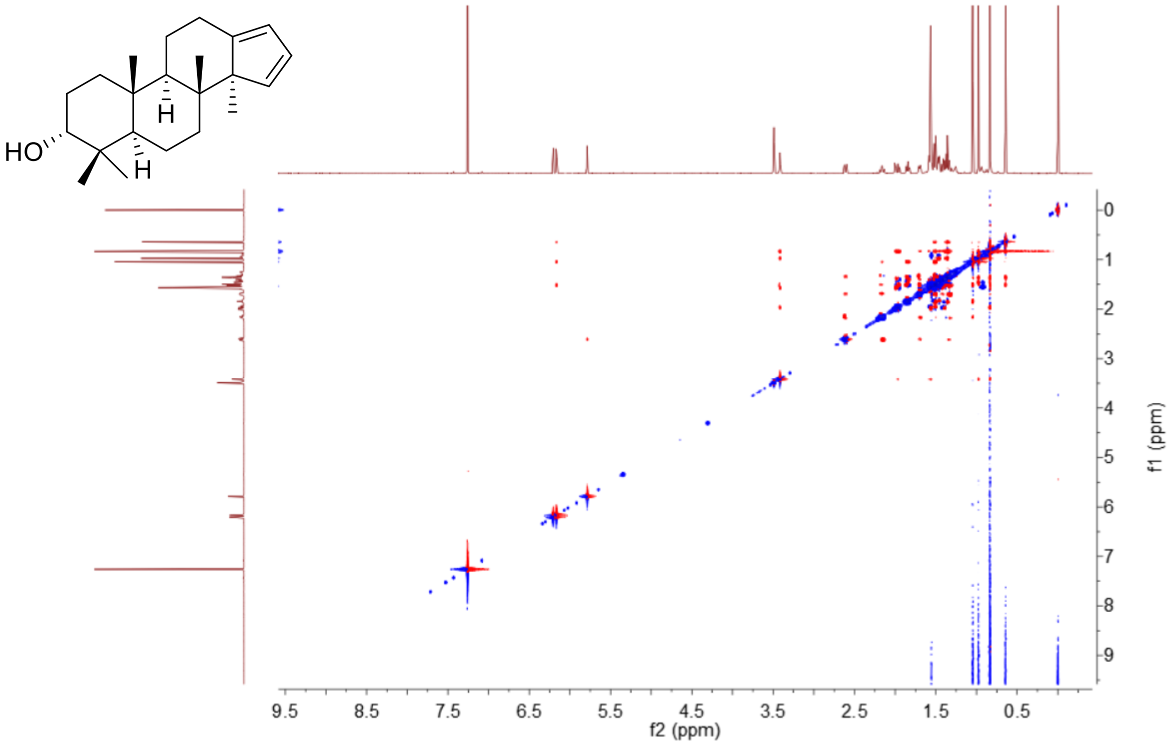

Figure S10. ROESY spectrum of **1** in $CDCl_3$.

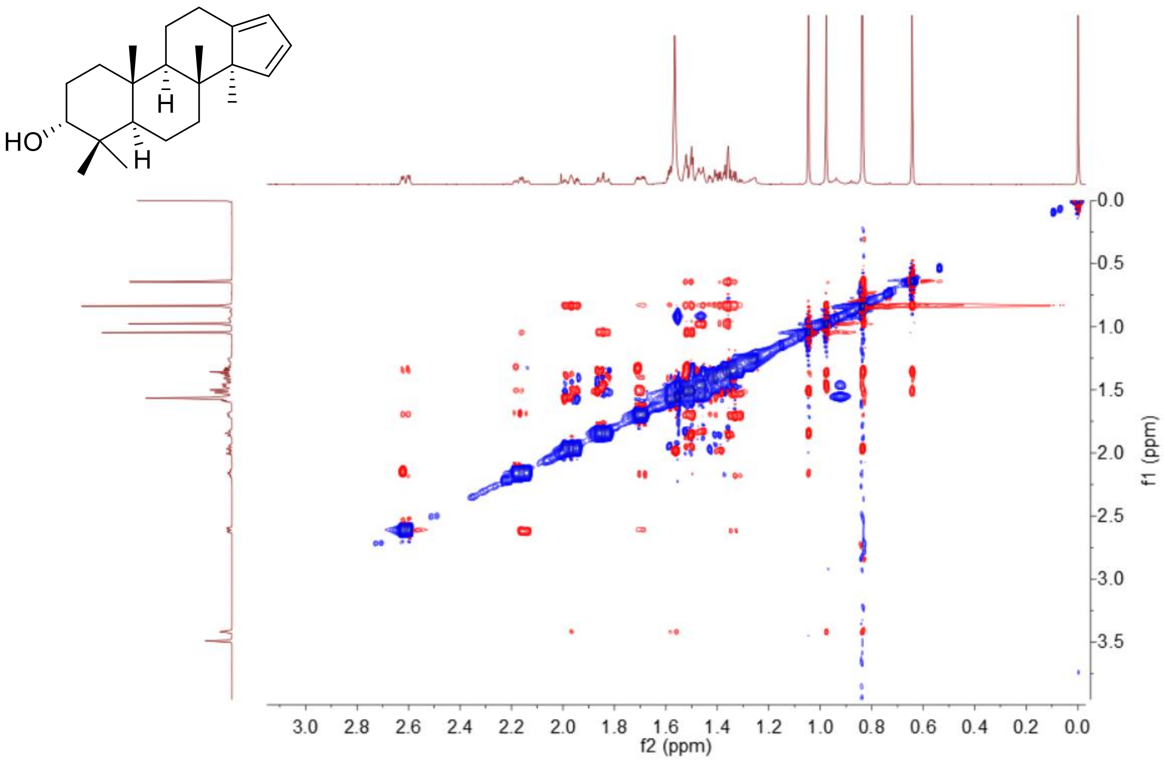

Figure S11. Enlarged ROESY spectrum of **1** in $CDCl_3$.

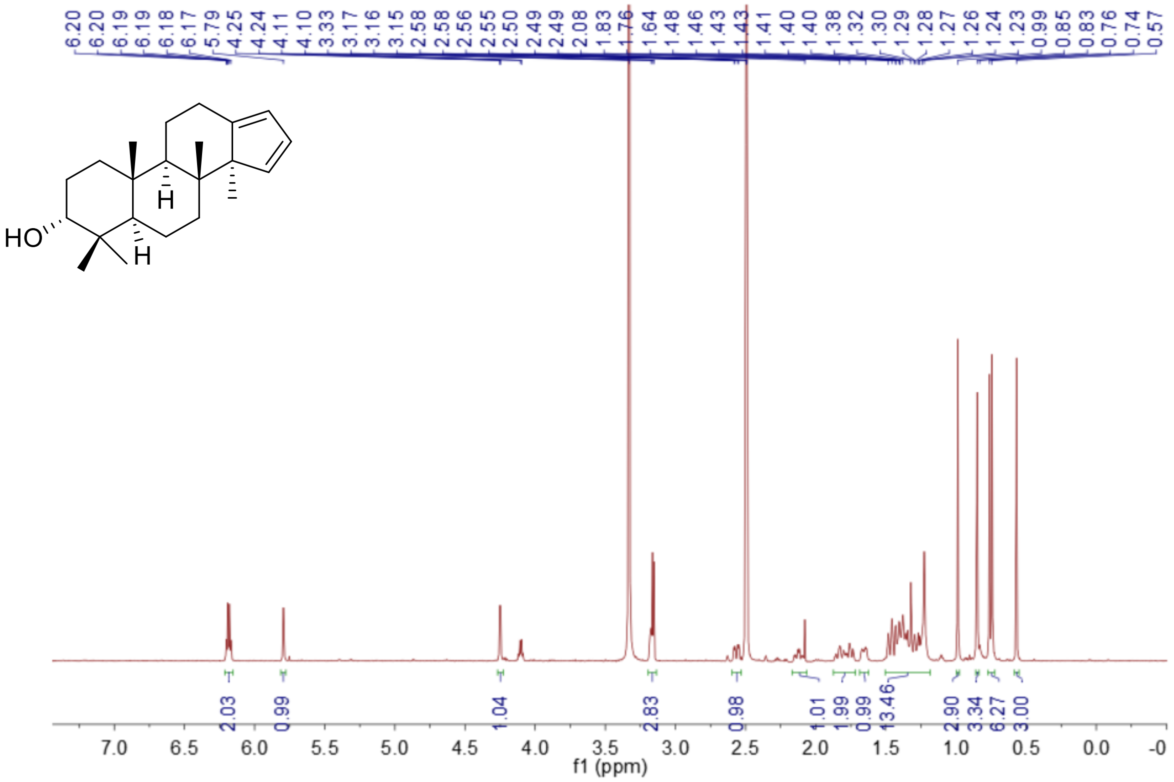

FIgure S12. ^{1}H NMR spectrum of **1** in DMSO-d_6.

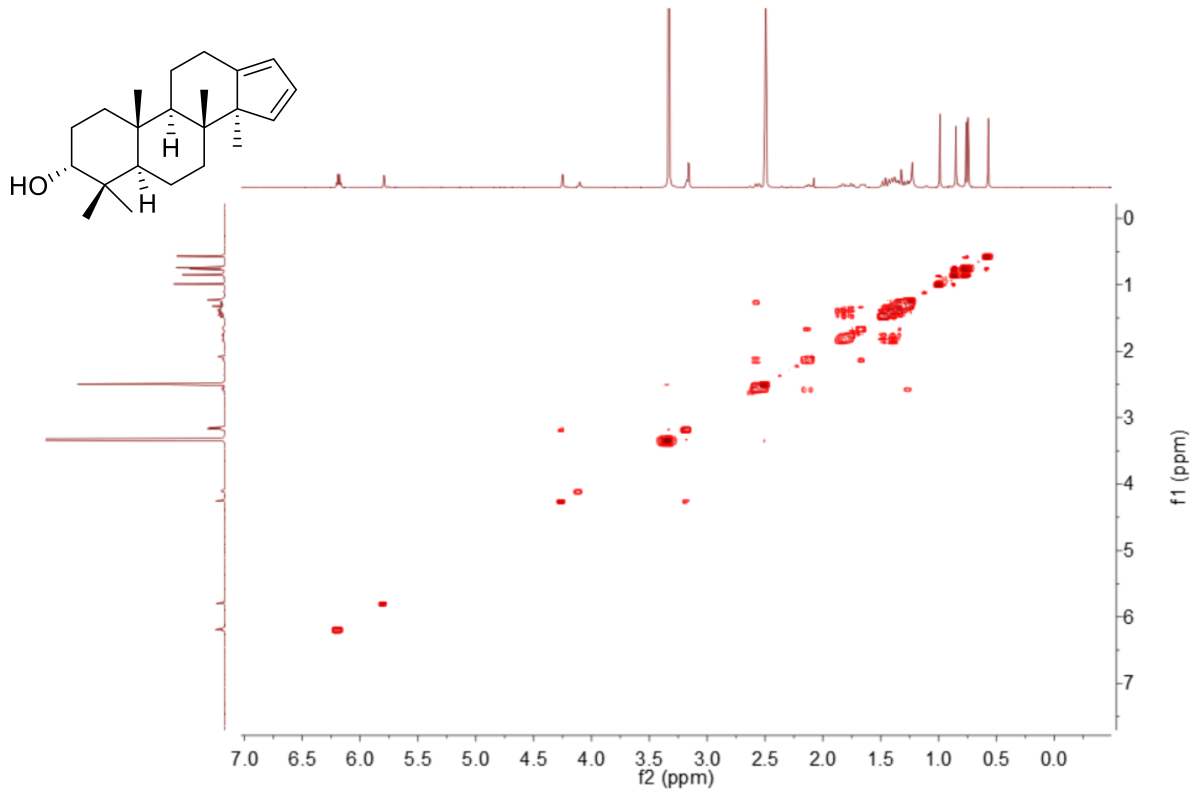

Figure S13. ^{1}H–^{1}H COSY spectrum of **1** in DMSO-d_6.

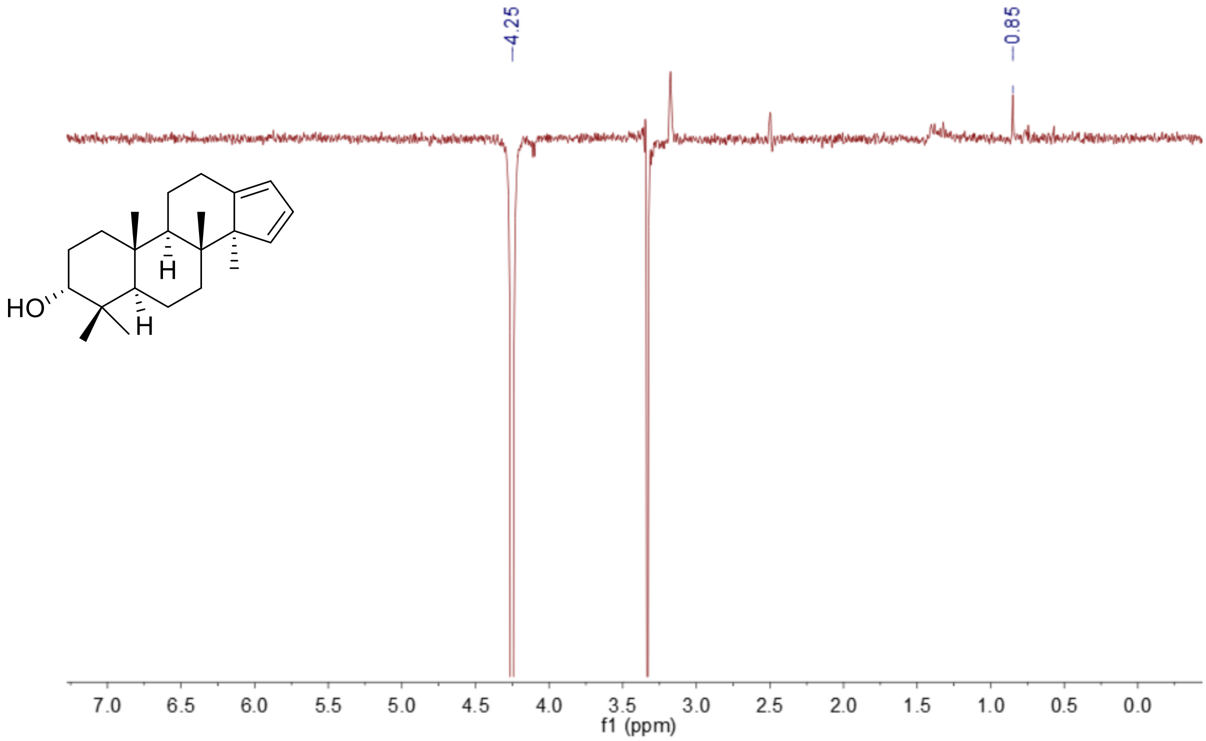

Figure S14. NOE spectrum of **1** in DMSO-d_6.

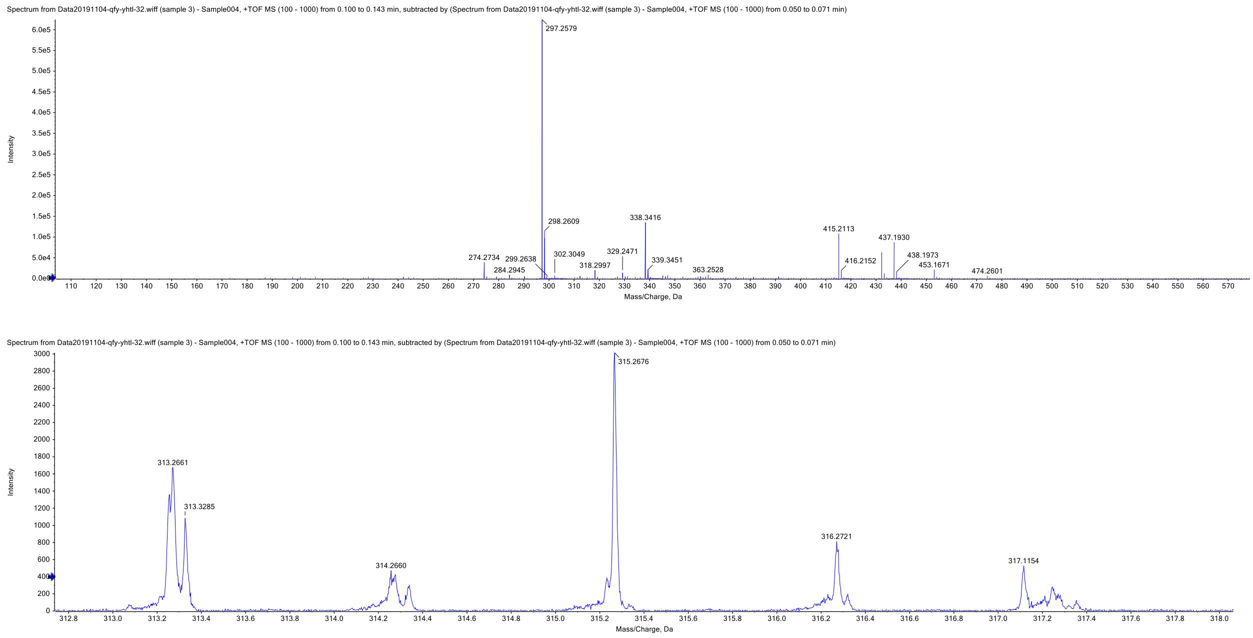

[M+H]+ m/z 315.2676

Hit	Formula	m/z	RDB	ppm
1	C22H34O	315.2682	6.0	-2.0

Elements from ~ to C22H34O

Mass tolerance 5 ppm

Figure S15. HRESIMS of **1.**

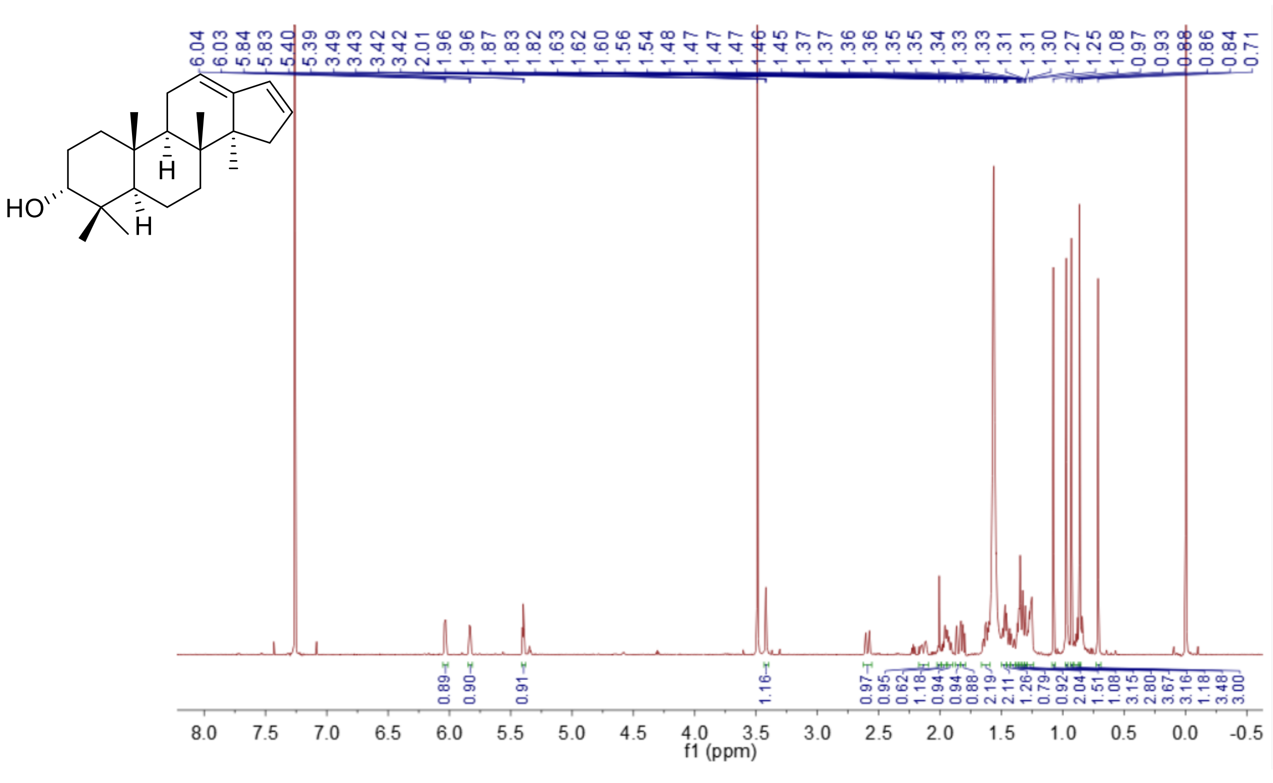

Figure S16. ^{1}H NMR spectrum of **2** in $CDCl_3$.

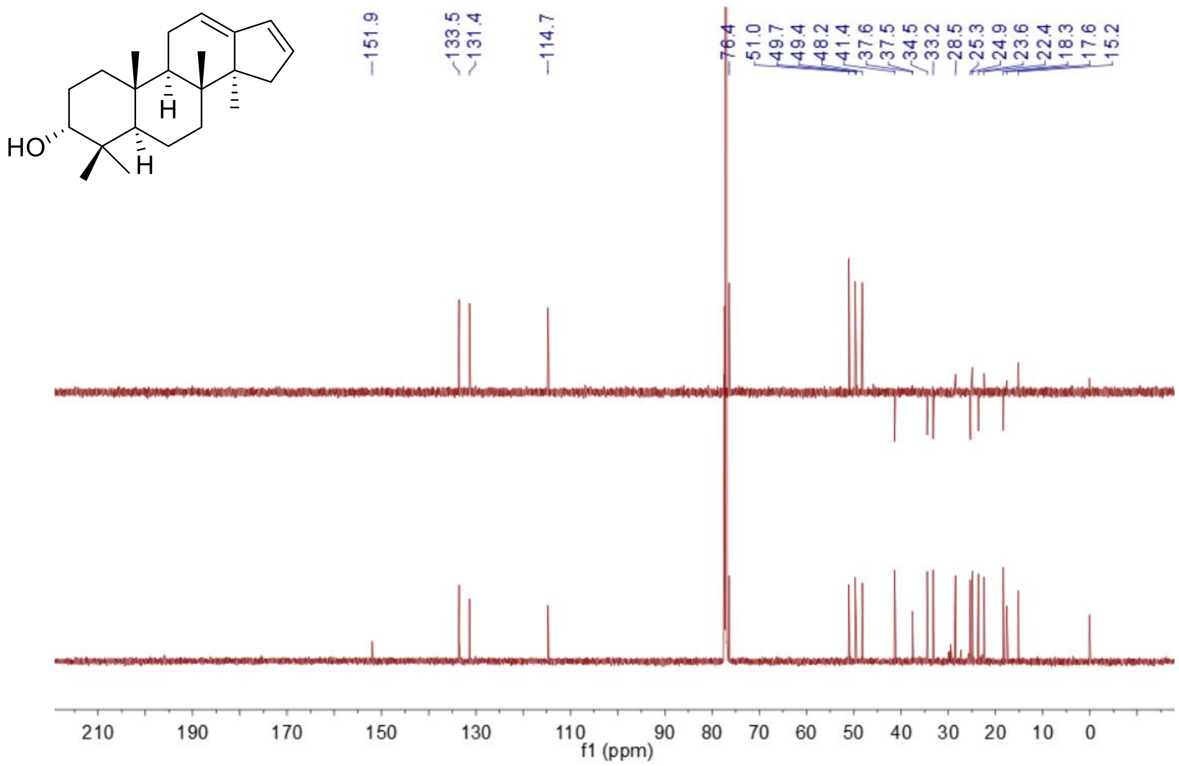

Figure S17. ^{13}C NMR and DEPT spectra of **2** in $CDCl_3$.

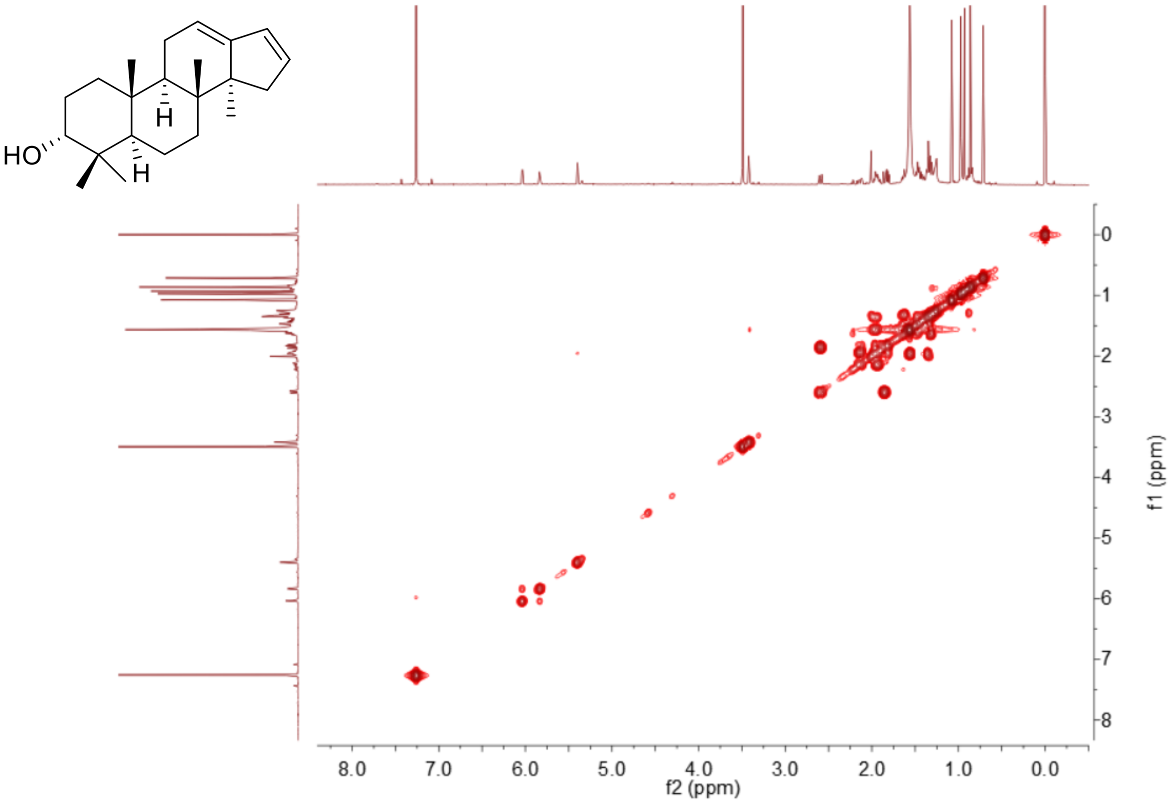

Figure S18. ^{1}H–^{1}H COSY spectrum of **2** in $CDCl_3$.

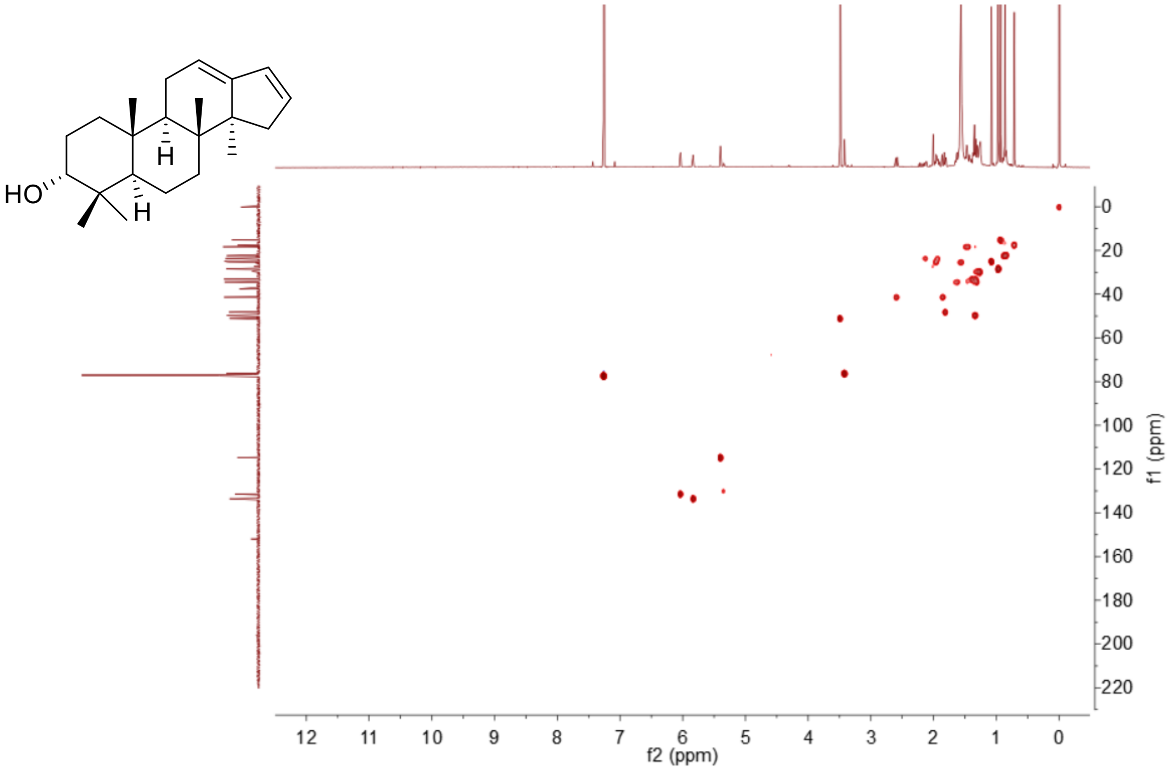

Figure S19. HSQC spectrum of **2** in $CDCl_3$.

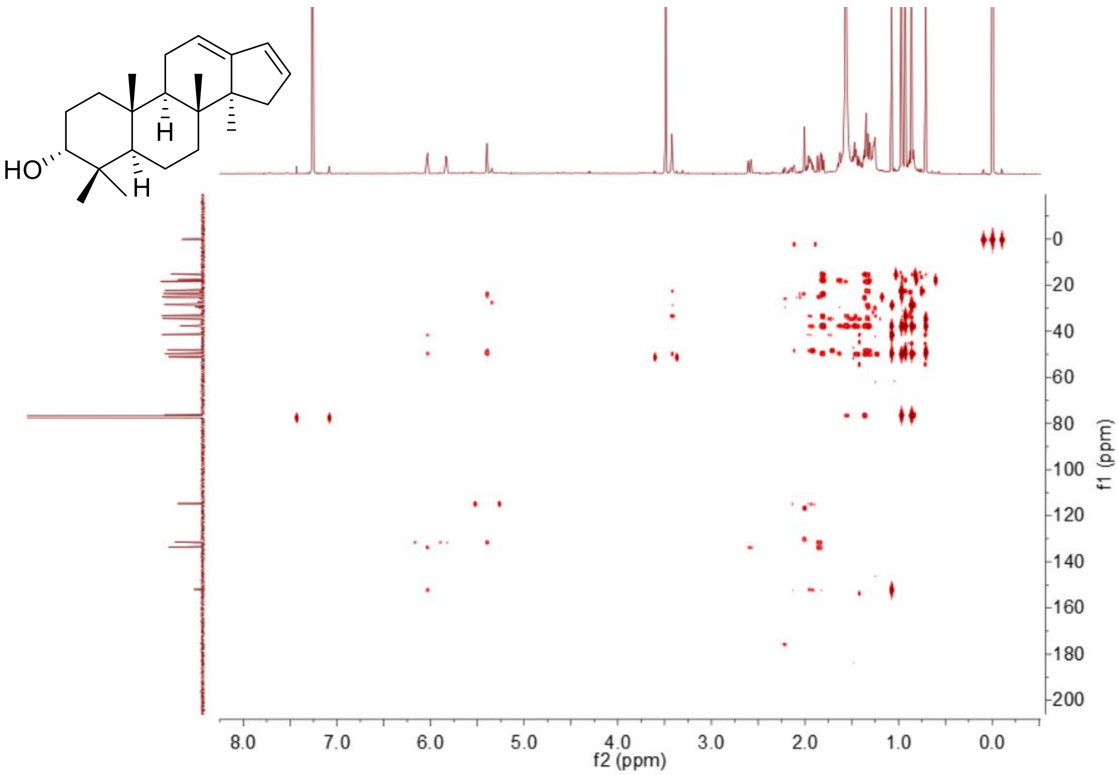

Figure S20. HMBC spectrum of **2** in $CDCl_3$.

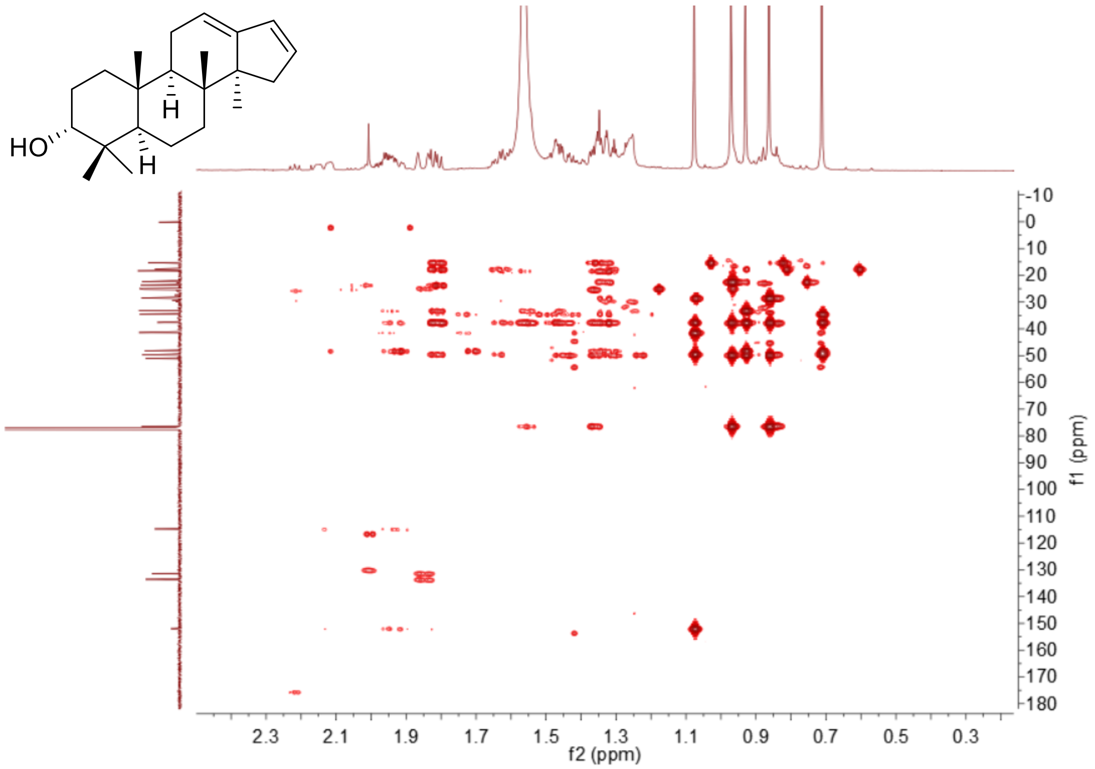

Figure S21. Enlarged HMBC spectrum of **2** in $CDCl_3$.

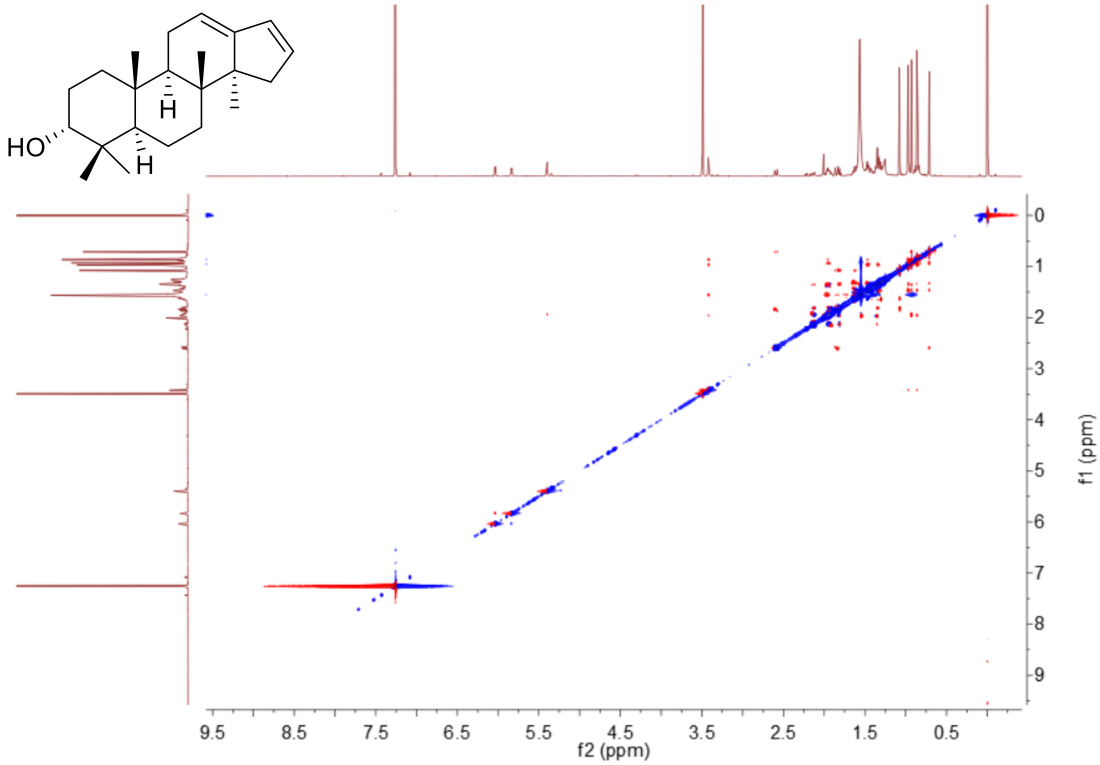

Figure S22. ROESY spectrum of **2** in $CDCl_3$.

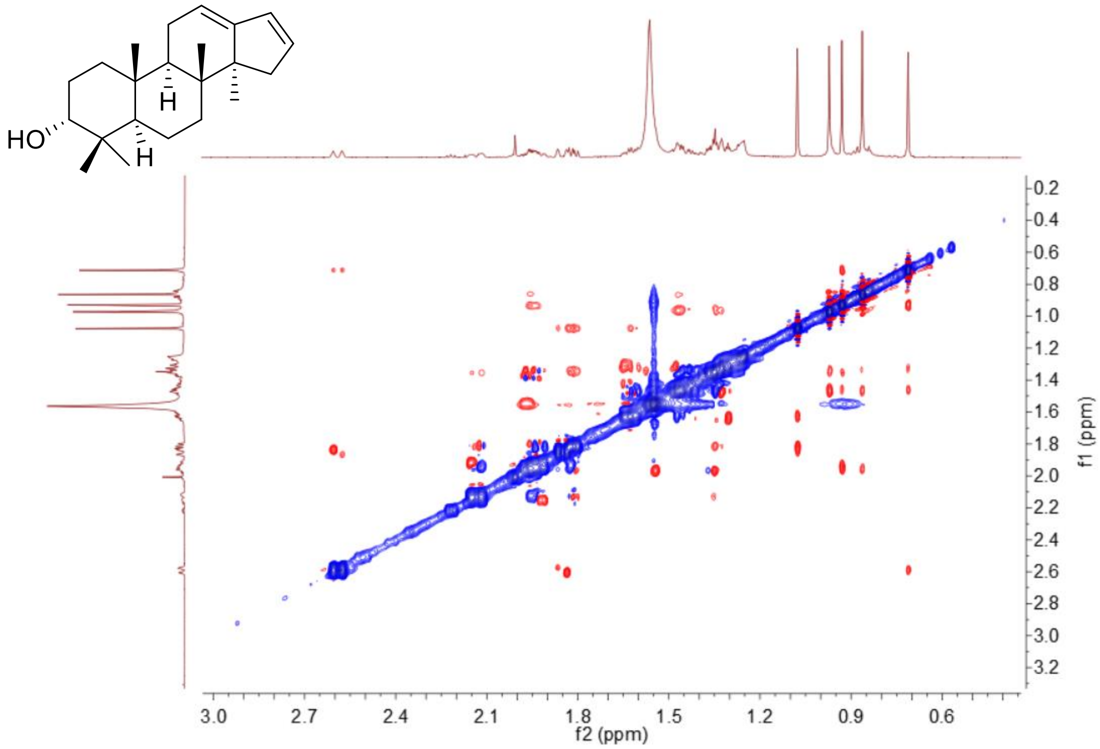

Figure S23. Enlarged ROESY spectrum of **2** in $CDCl_3$.

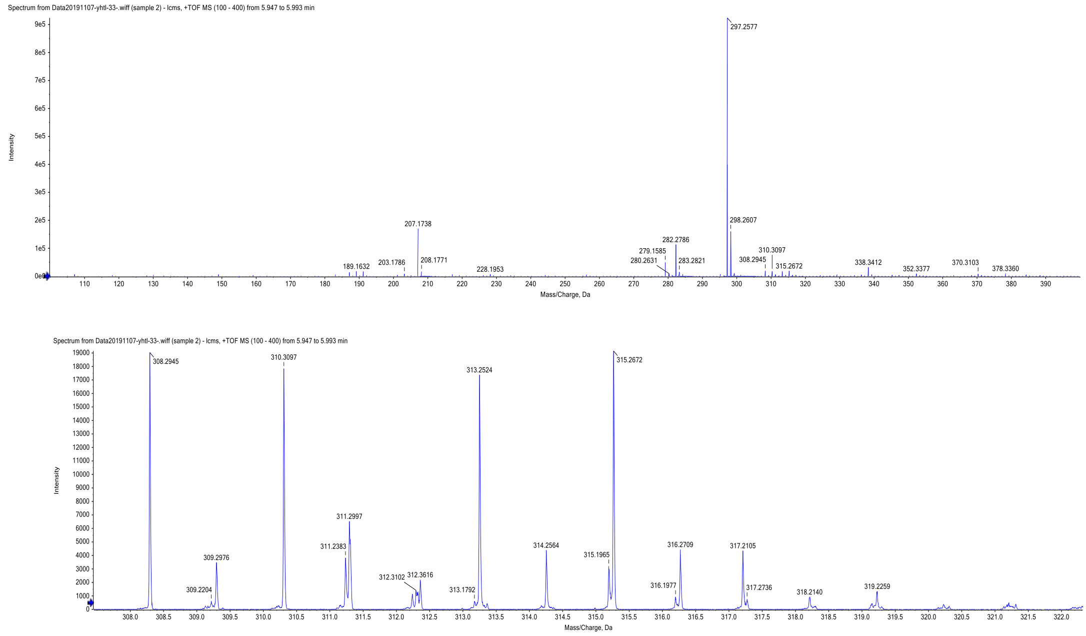

[M+H]+ m/z 315.2672

Hit	Formula	m/z	RDB	ppm
1	C22H34O	315.2682	6.0	-3.3

Elements from ~ to C22H34O

Mass tolerance 5 ppm

Figure S24. HRESIMS of **2**.

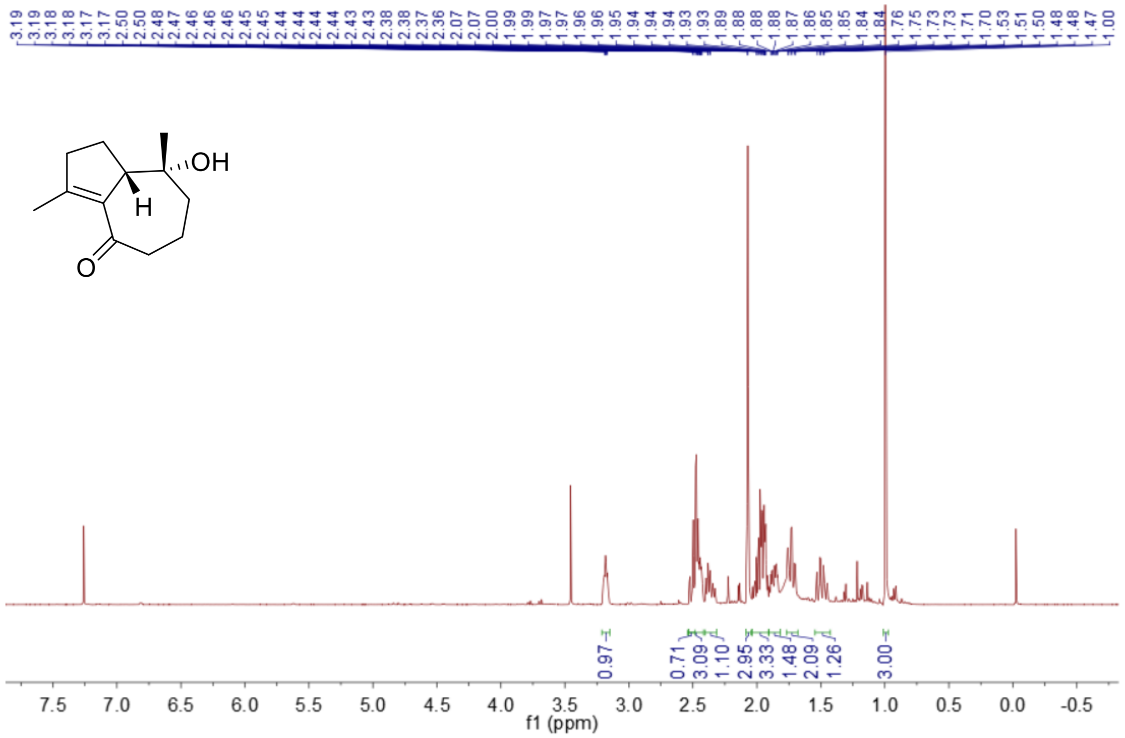

Figure S25. ^{1}H NMR spectrum of **3** in $CDCl_3$.

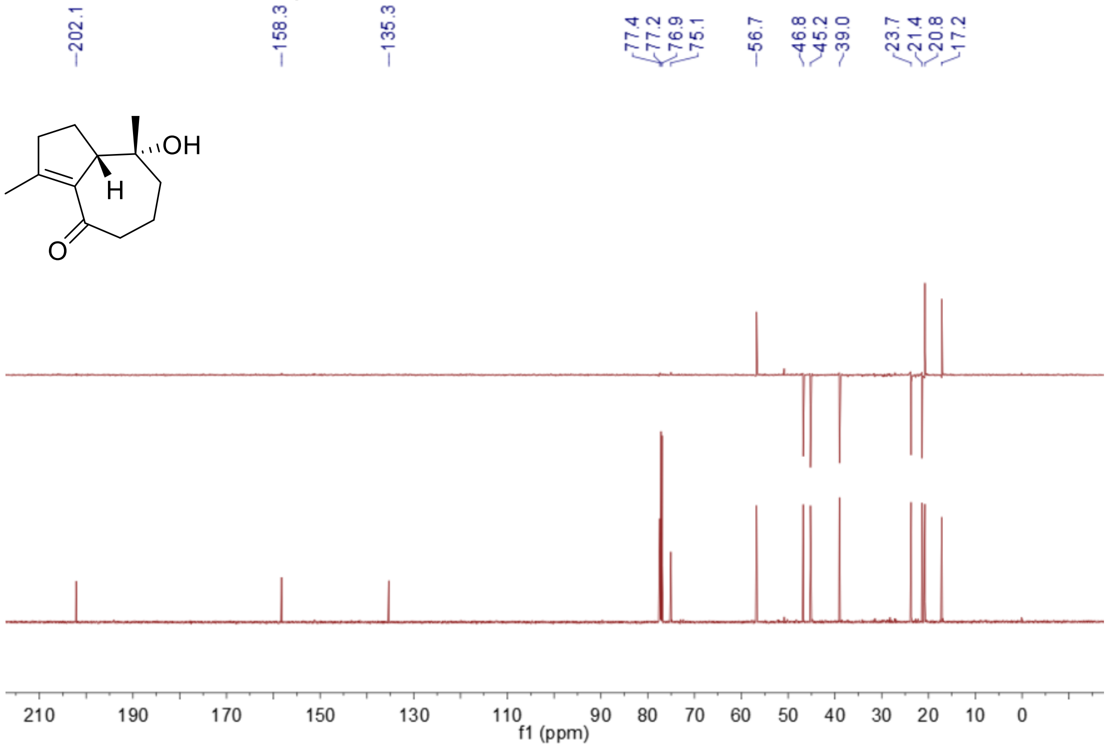

Figure S26. ^{13}C NMR and DEPT spectra of **3** in $CDCl_3$.

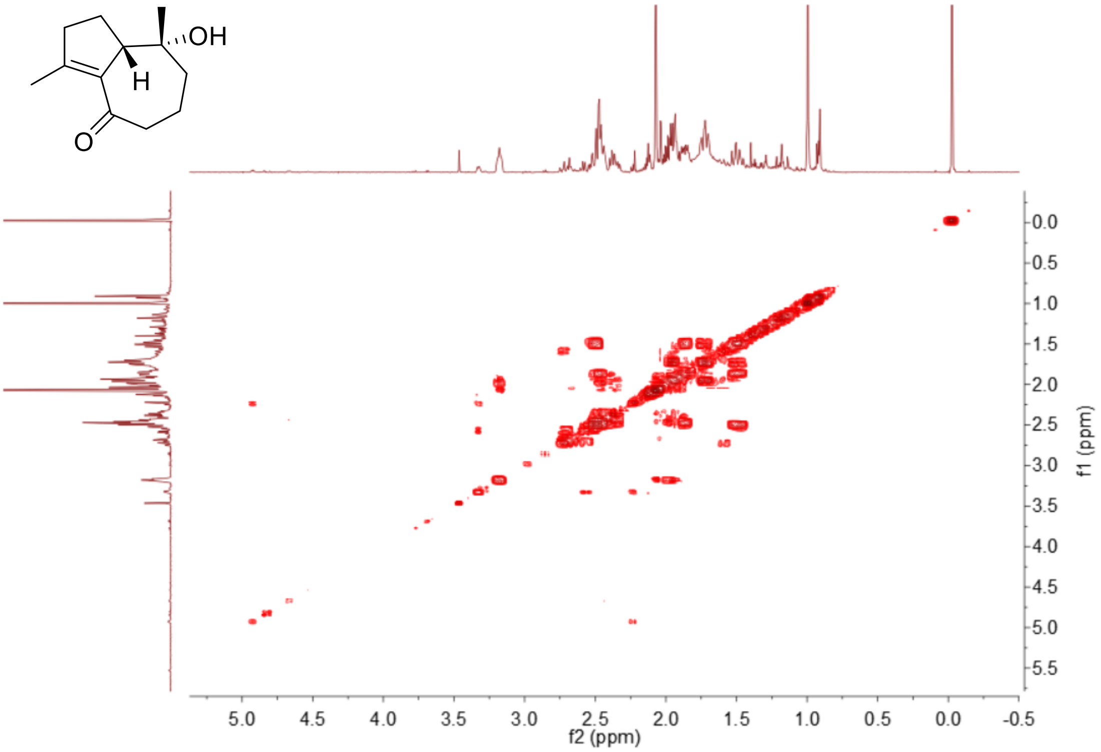

Figure S27. ^1H–^1H COSY spectra of **3** in $CDCl_3$.

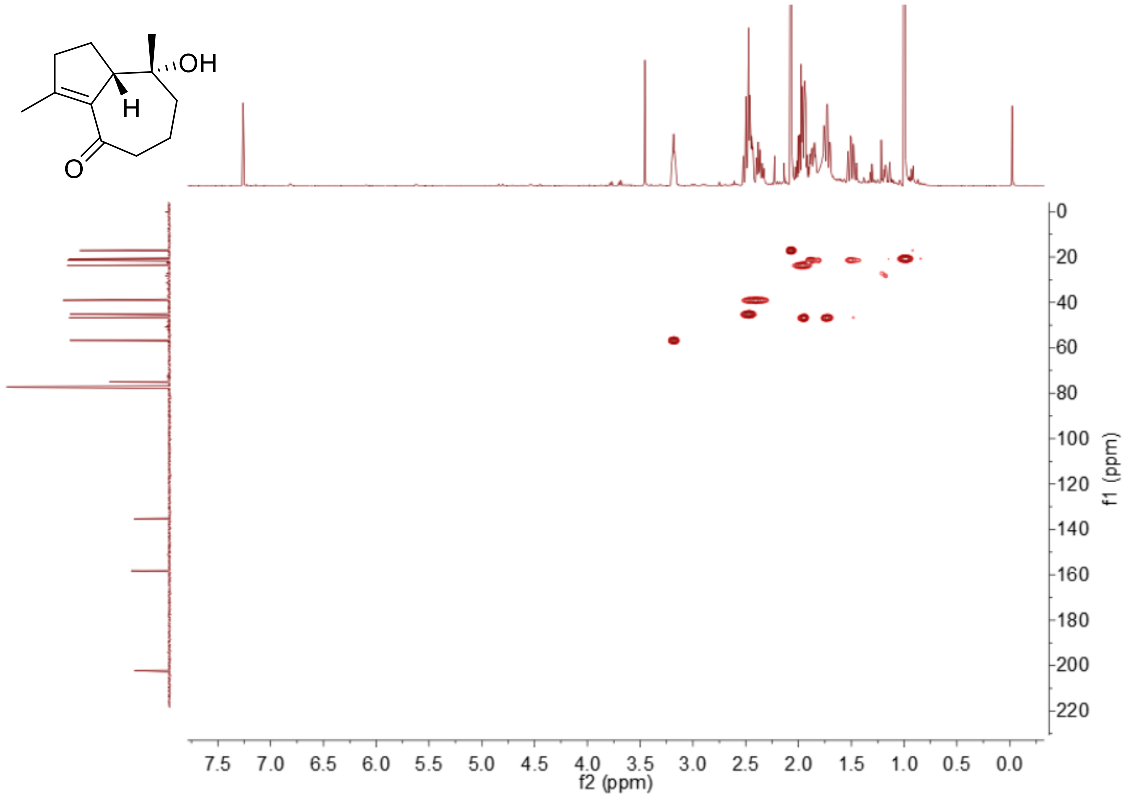

Figure S28. HSQC spectrum of **3** in $CDCl_3$.

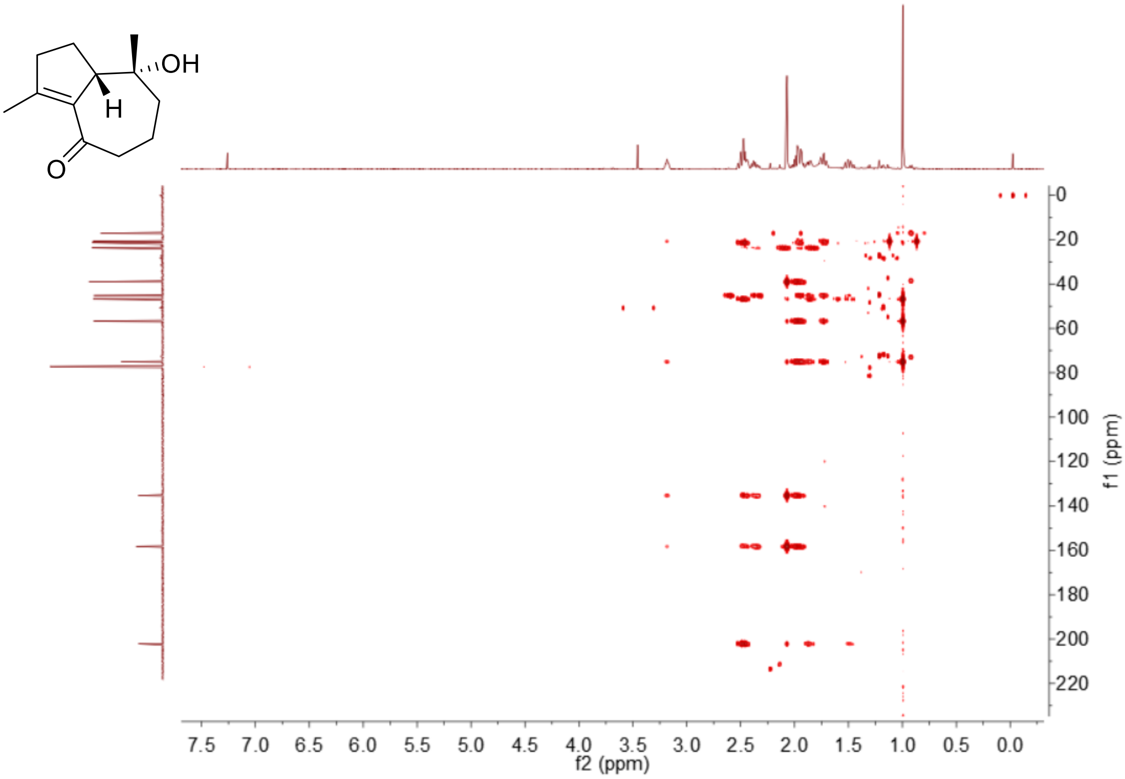

Figure S29. HMBC spectrum of **3** in $CDCl_3$.

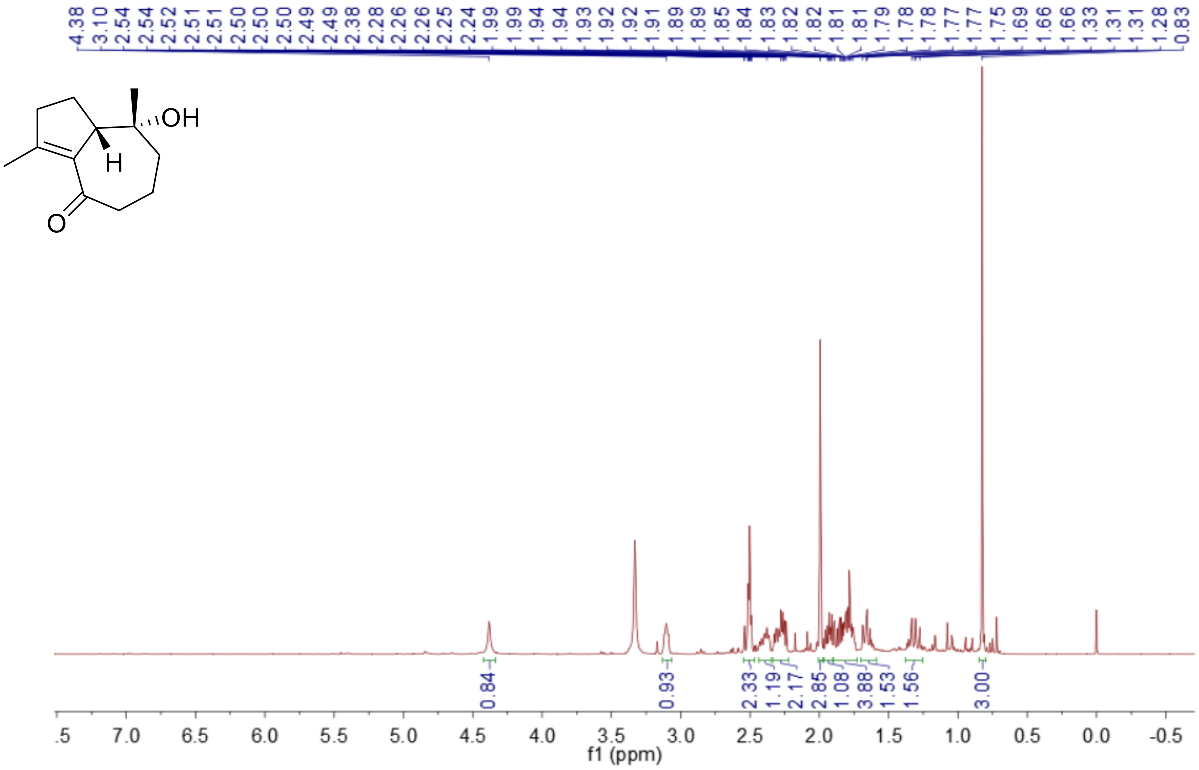

Figure S30. ^{1}H NMR spectrum of **3** in DMSO-d_6.

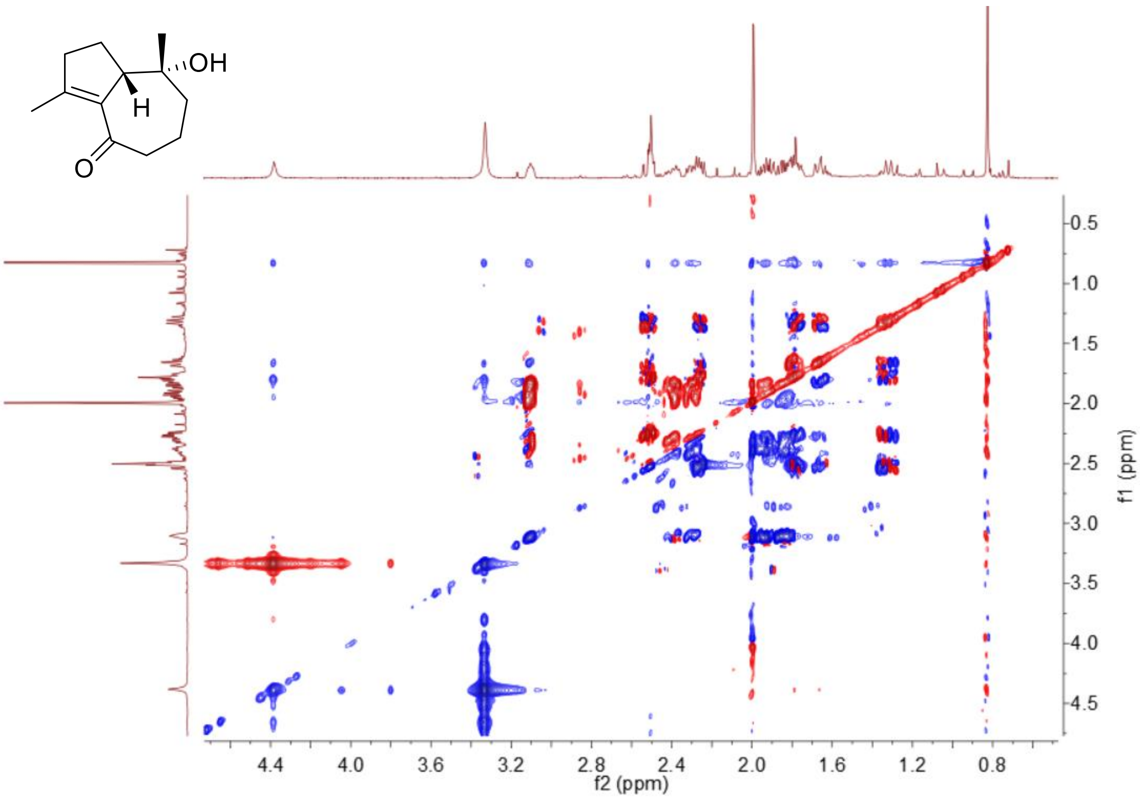

Figure S31. ROESY spectrum of **3** in DMSO-d_6.

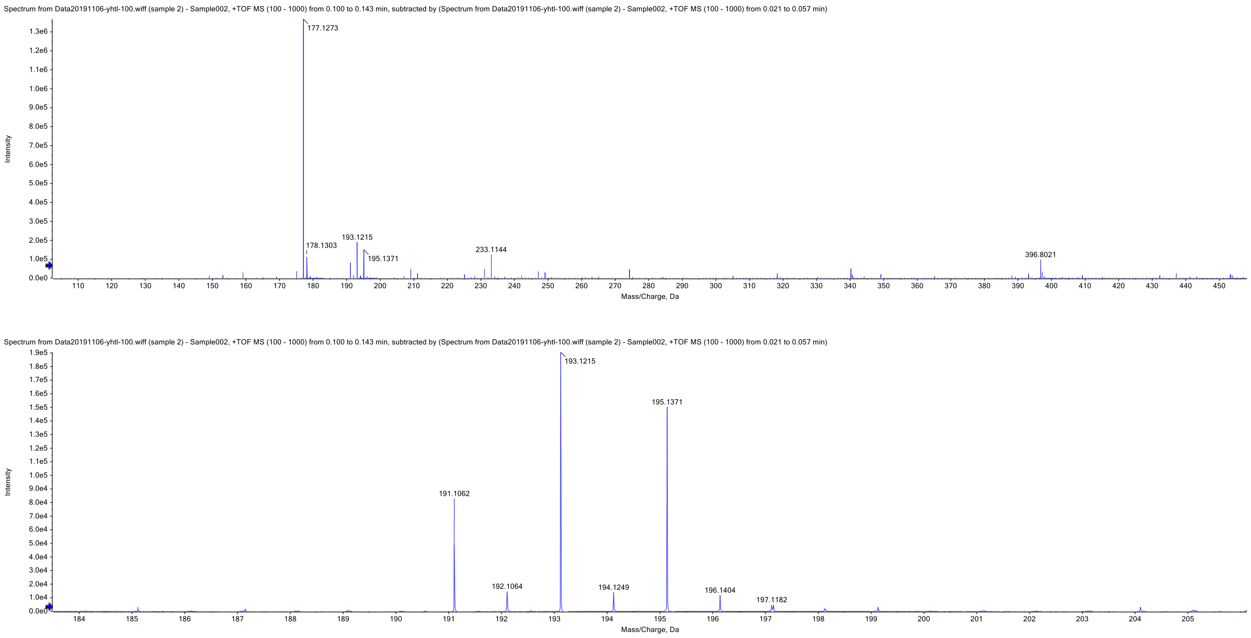

$[M+H]^+$ m/z 195.1371

Hit	Formula	m/z	RDB	ppm
1	C12H18O2	195.1380	4.0	-4.4

Elements from ~ to C12H18O2

Mass tolerance 5 ppm

Figure S32. HRESIMS of **3**.

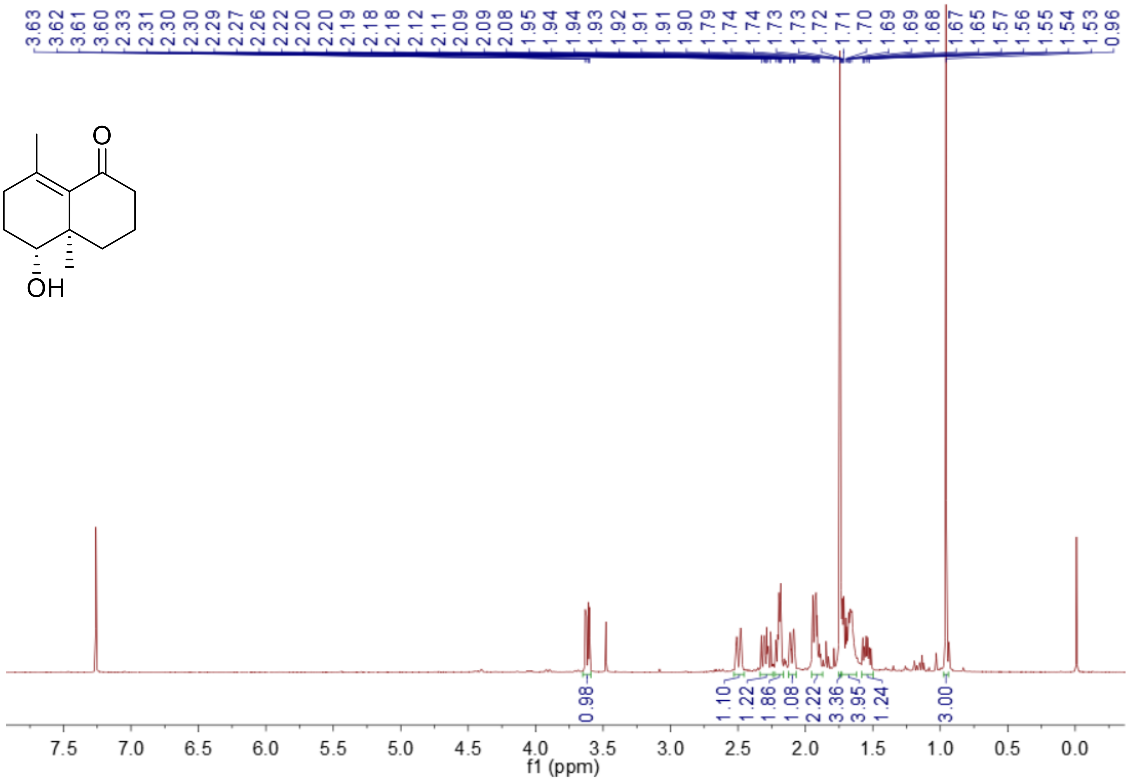

Figure S33. ^{1}H NMR spectrum of **4** in $CDCl_3$.

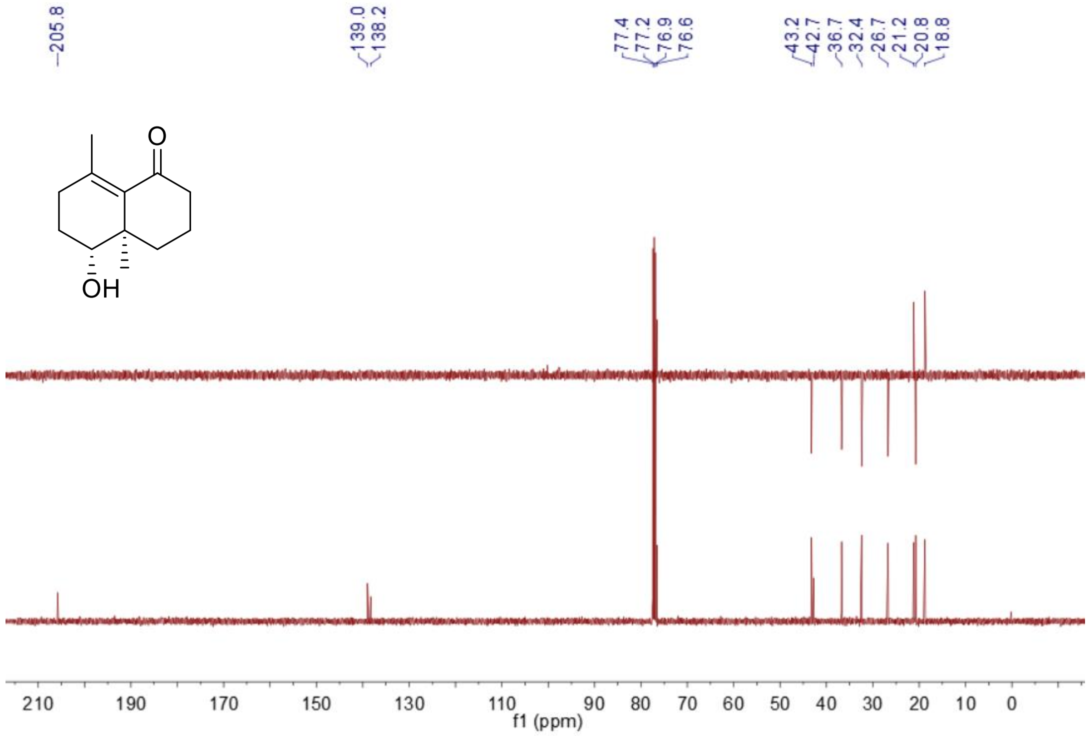

Figure S34. ^{13}C NMR and DEPT spectra of **4** in $CDCl_3$.

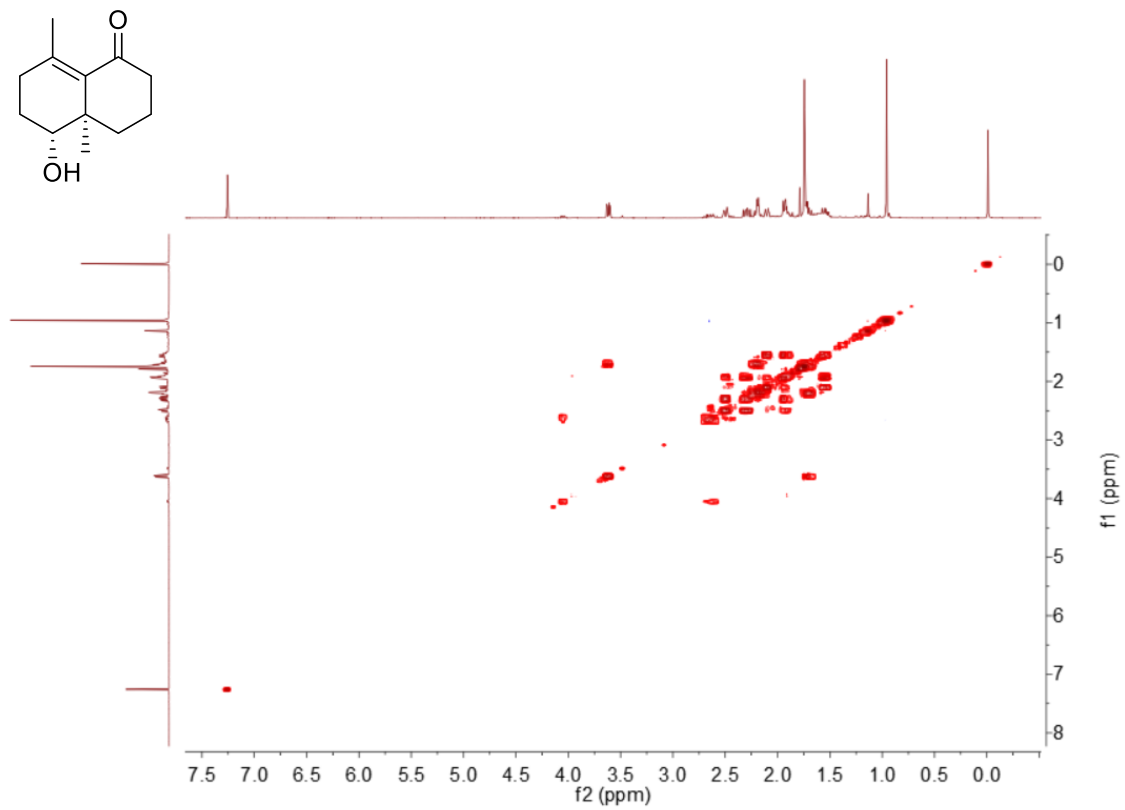

Figure S35. ^{1}H–^{1}H COSY spectrum of **4** in $CDCl_3$.

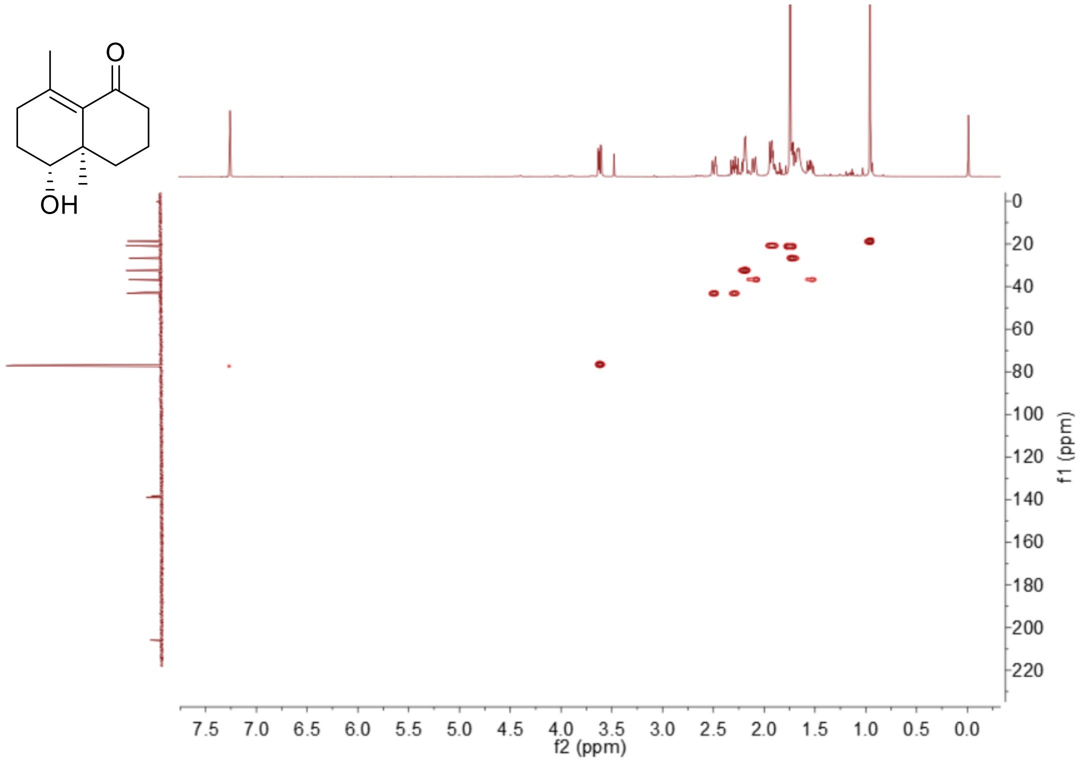

Figure S36. HSQC spectrum of **4** in $CDCl_3$.

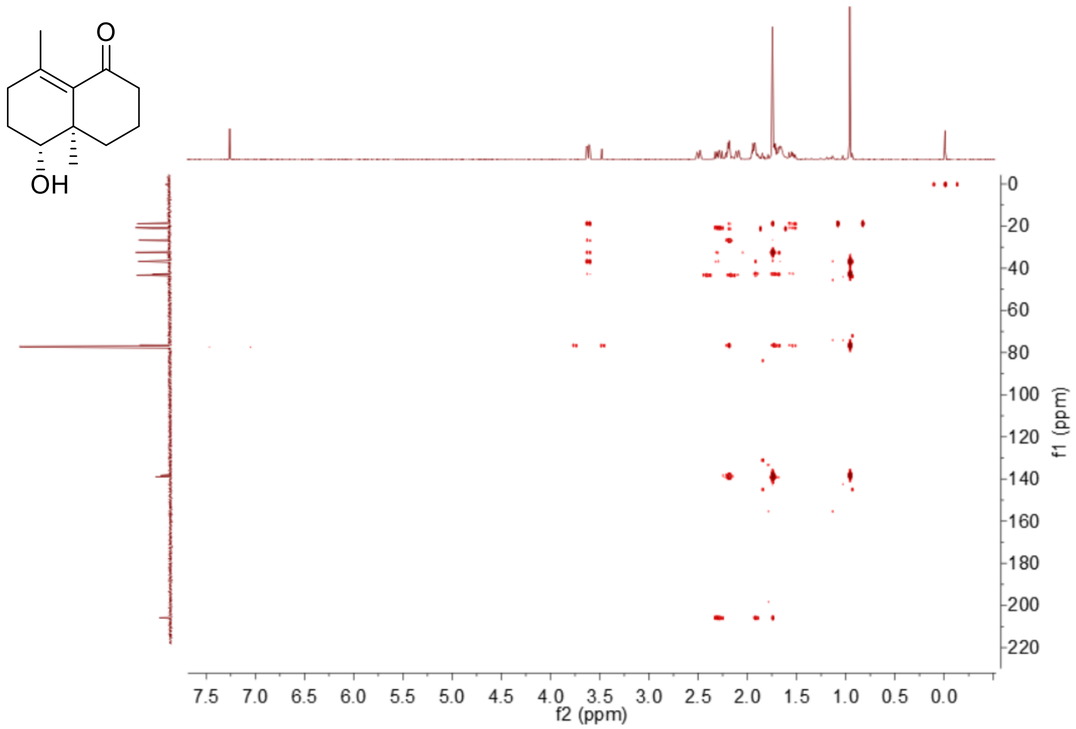

Figure S37. HMBC spectrum of **4** in $CDCl_3$.

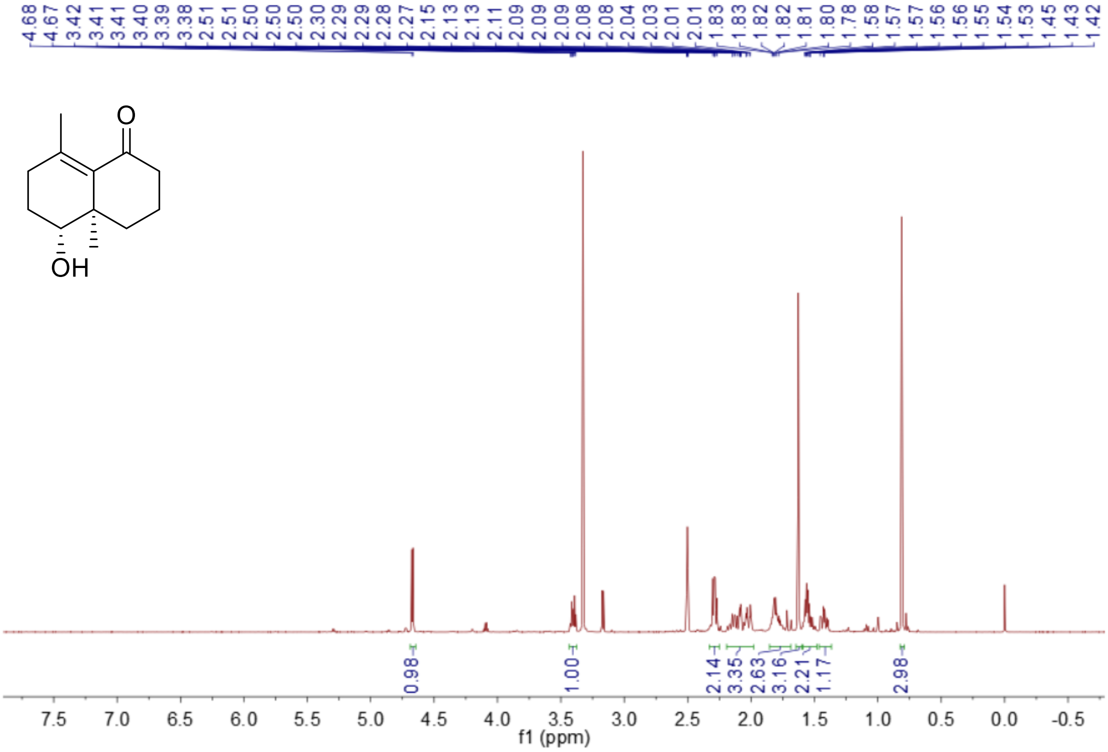

Figure S38. ^{1}H NMR spectrum of **4** in DMSO-d_6.

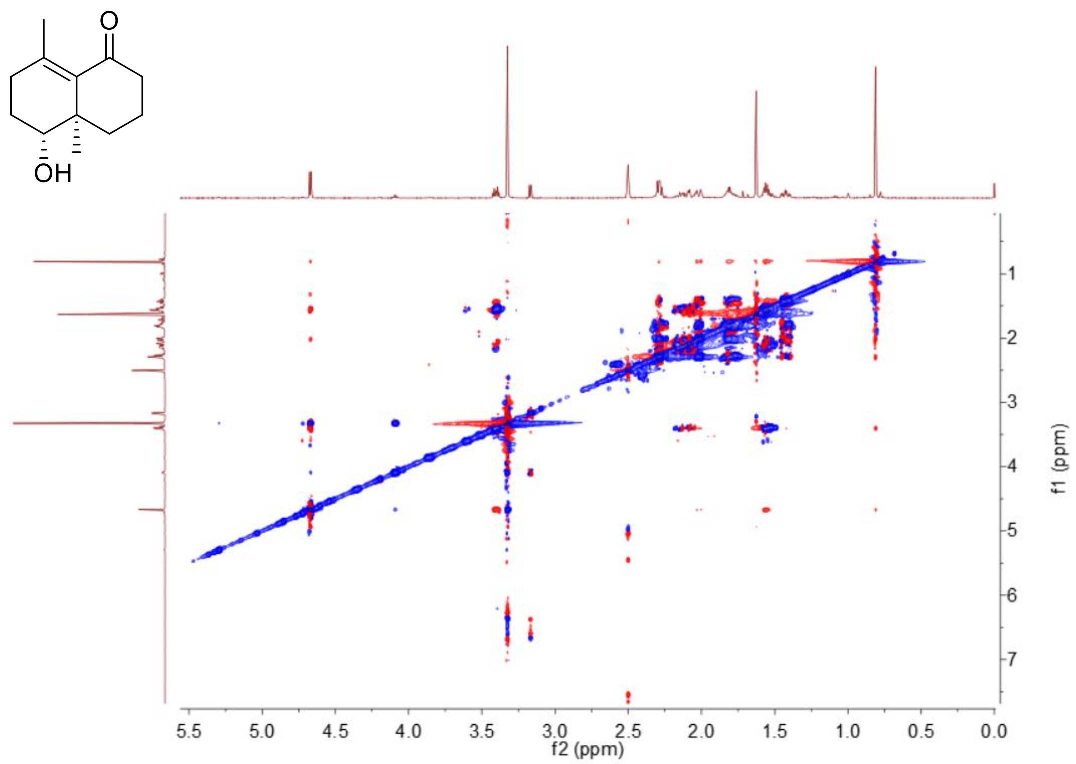

Figure S39. ROESY spectrum of **4** in DMSO-d_6.

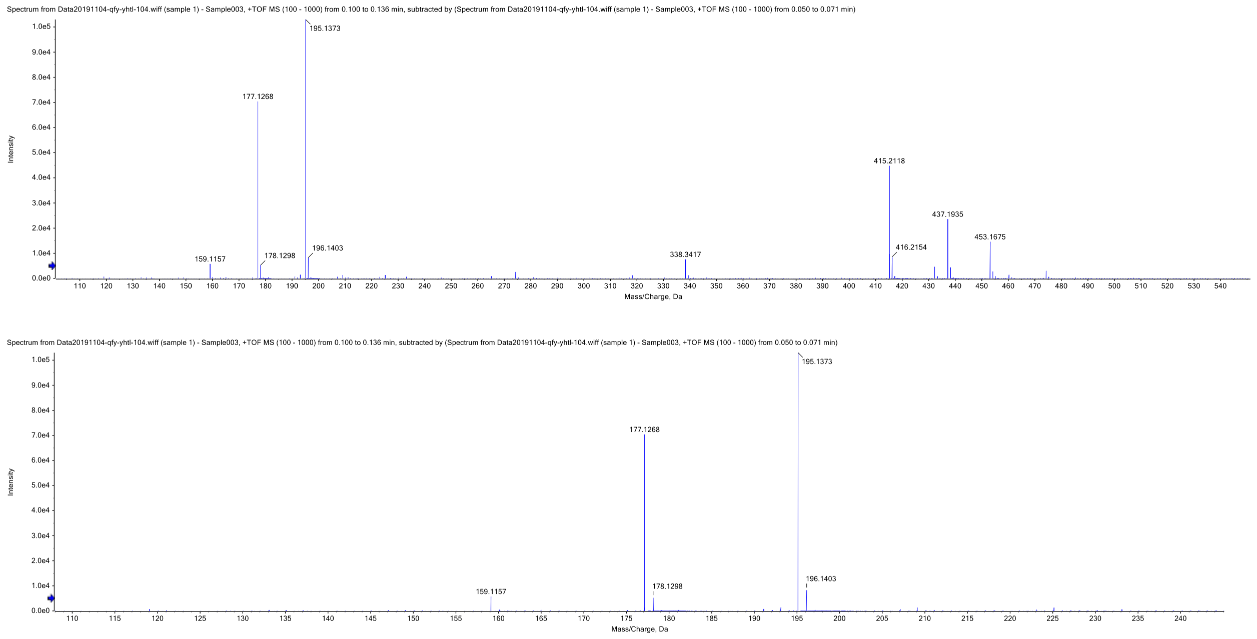

$[M+H]^+$ m/z 195.1373

Hit	Formula	m/z	RDB	ppm
1	C12H18O2	195.1380	4.0	-3.4

Elements from ~ to C12H18O2

Mass tolerance 5 ppm

Figure S40. HRESIMS of **4.**

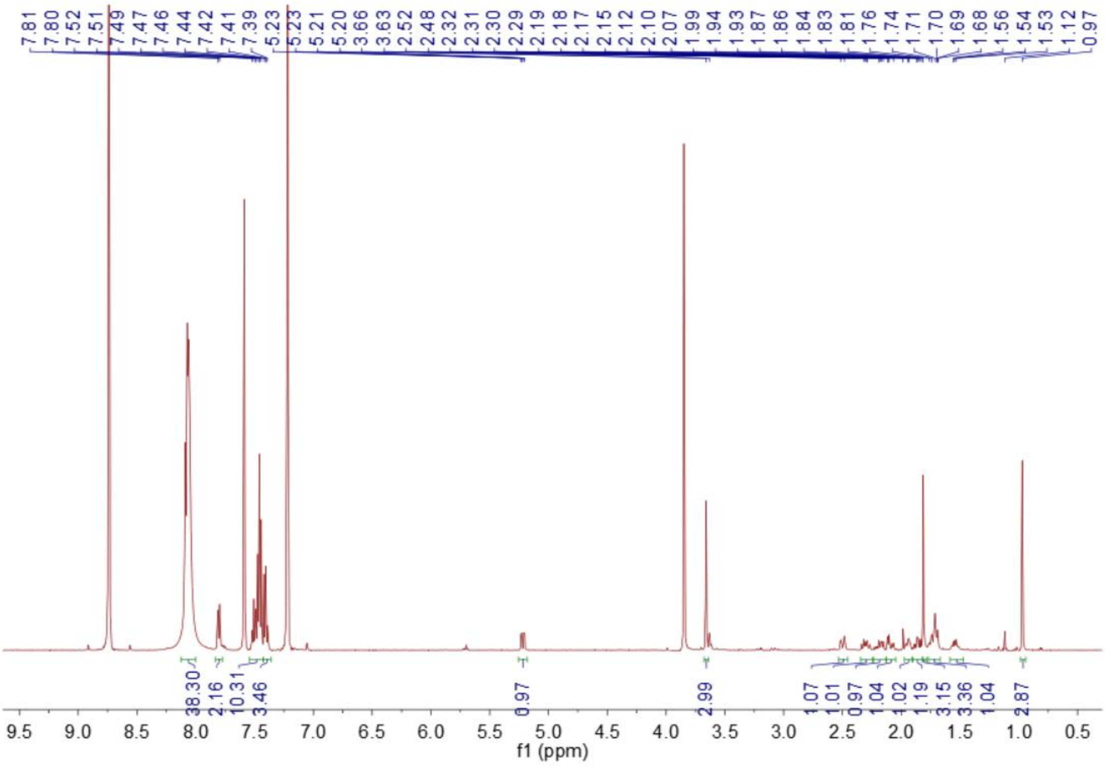

Figure S41. ^{1}H NMR spectrum of **4**a in pyridine.

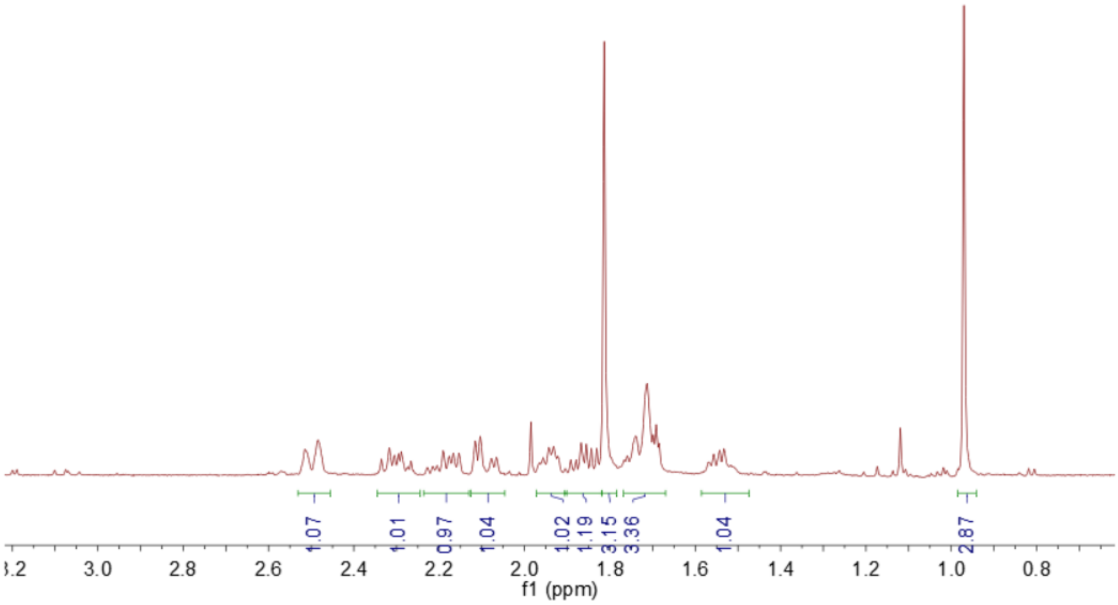

Figure S42. Enlarged ^{1}H NMR spectrum of **4**a in pyridine.

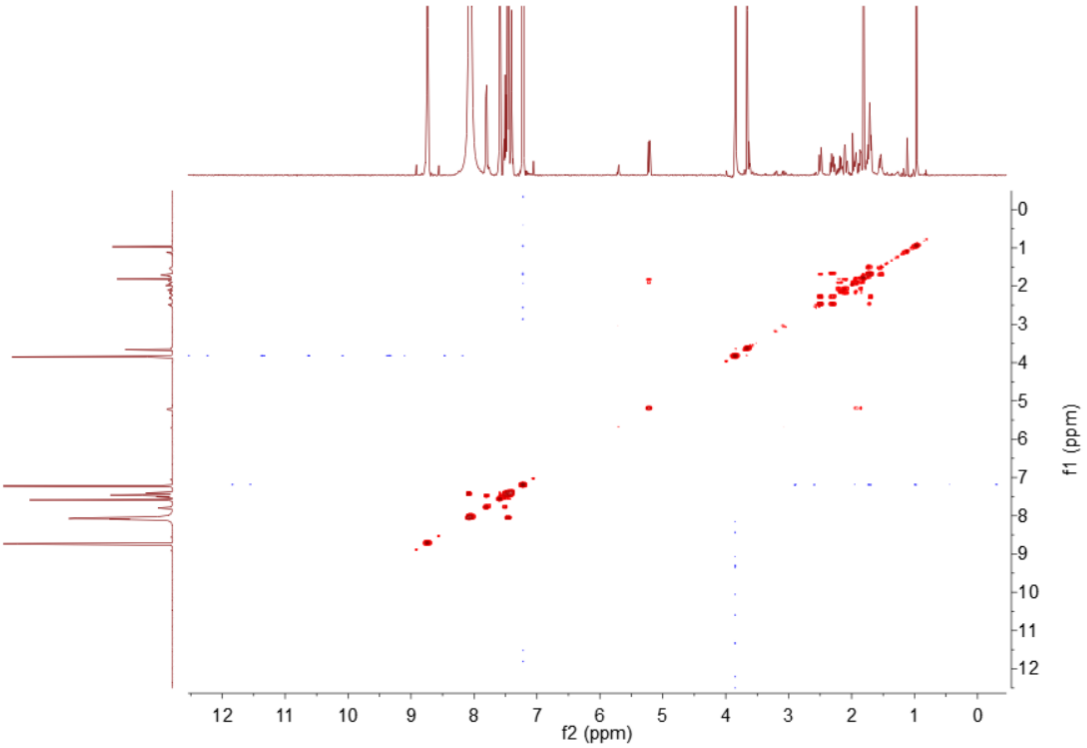

Figure S43. ^1H–^1H COSY spectrum of **4**a in pyridine.

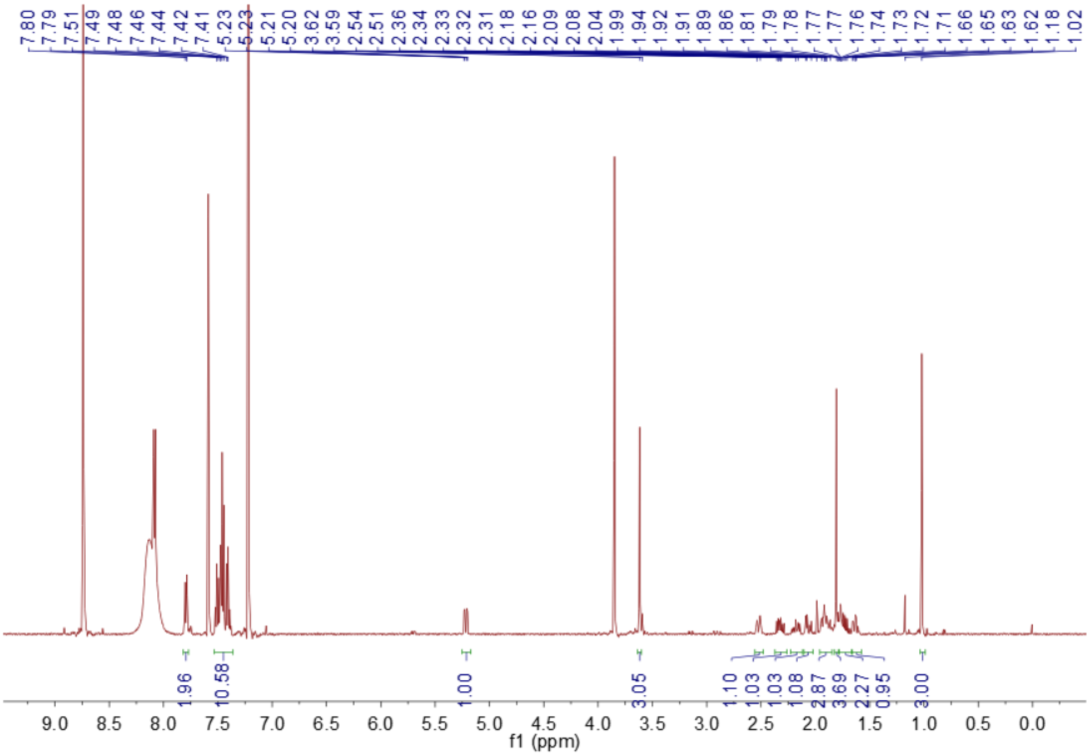

Figure S44. ^1H NMR spectrum of **4**b in pyridine.

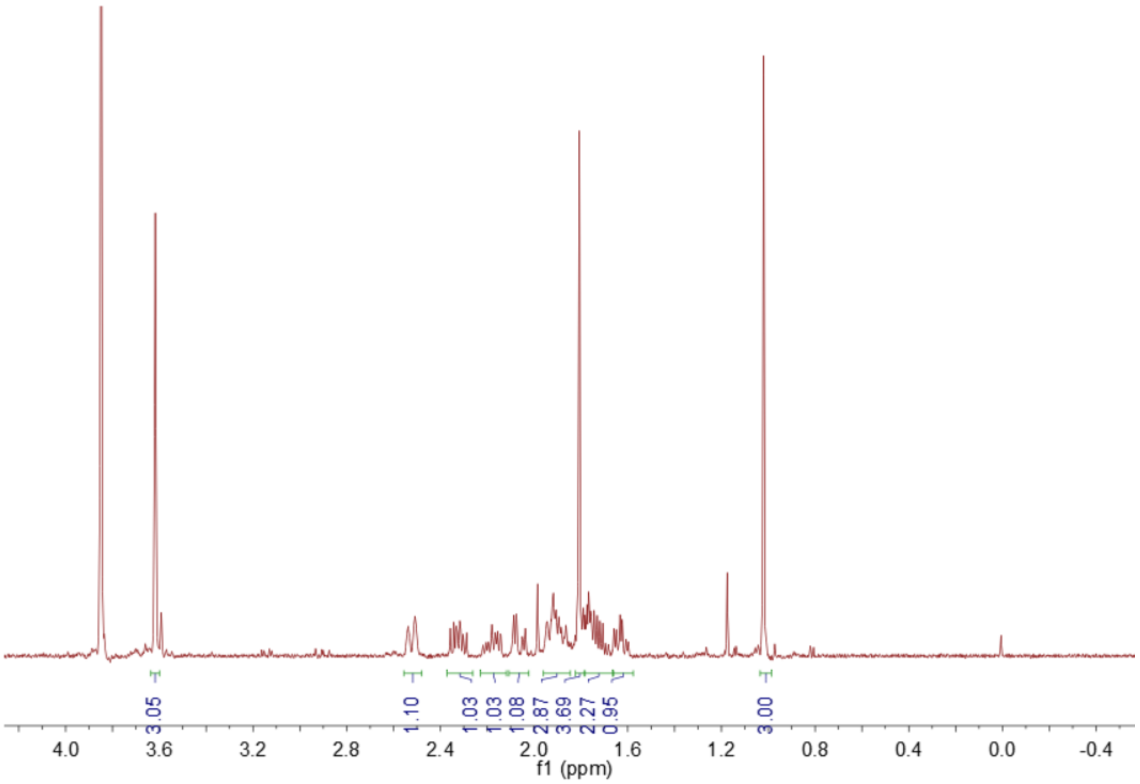

Figure S45. Enlarged ^1H NMR spectrum of **4**b in pyridine.

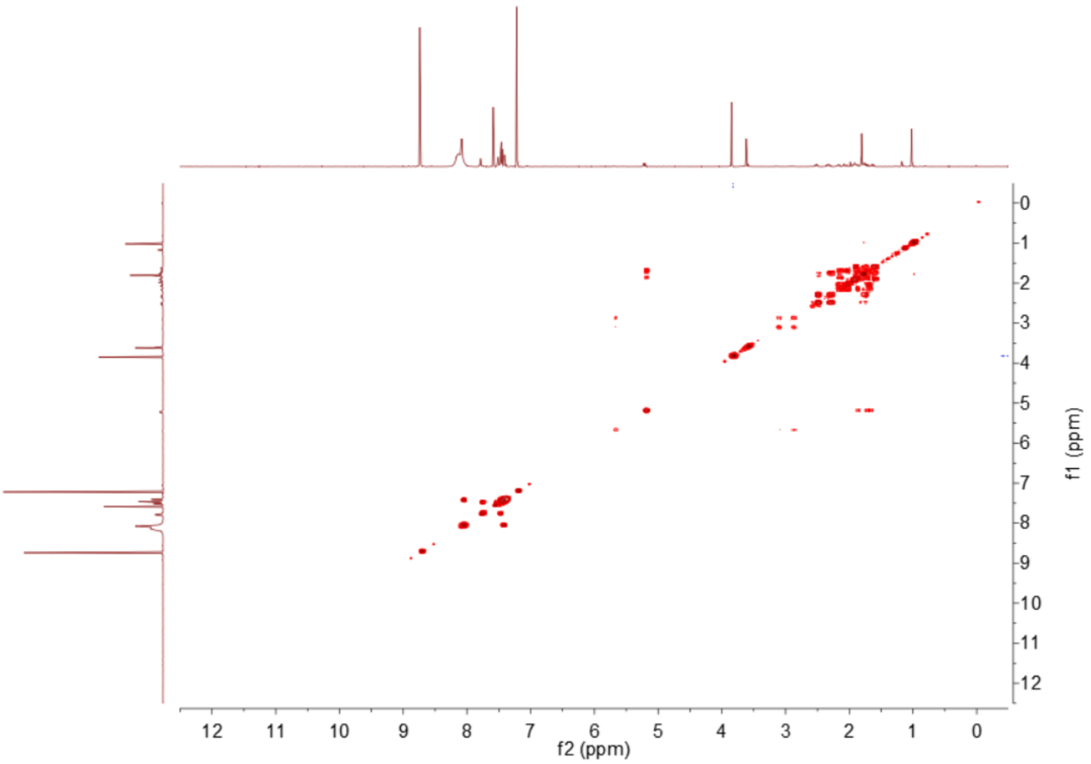

Figure S46. ^1H–^1H COSY spectrum of **4b** in pyridine.

ECD calculation for compounds **1–4**

Conformation search using molecular mechanics calculations was performed in CONFLEX version 7.0 with MMFF force field with an energy window for acceptable conformers (ewindow) of 5 kcal/mol above the ground state, a maximum number of conformations per molecule (maxconfs) of 100, and an RMSD cutoff (rmsd) of 0.5Å. Then the predominant conformers were optimized at B3LYP/6-311+G(d) level in Gaussian 09 (Frisch et al. 2009)[1]. The optimized conformation geometries and thermodynamic parameters of all selected conformations were provided. The optimized conformers of **1**–**4** were used for the ECD calculation, which were performed with Gaussian 09 (B3LYP/6-311+G(d)). The solvent effects were taken into account by the polarizable-conductor calculation model (PCM, methanol as the solvent). Percentages for each conformation are shown in Table S1.

Selected conformation of 1 and their percentage

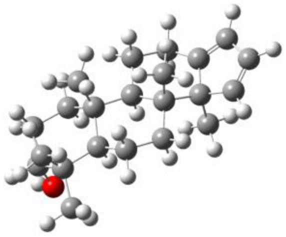

1-1 (34.75%)

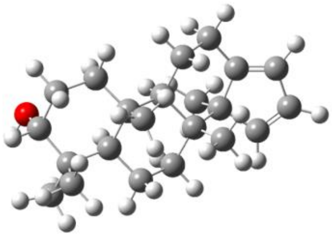

1-2 (65.25%)

Figure S47. The lowest energy conformers of **1** (the relative populations are in parentheses).

Selected conformation of 2 and their percentage

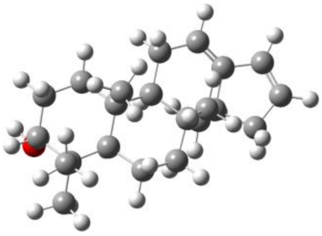

2-1 (29.52%)

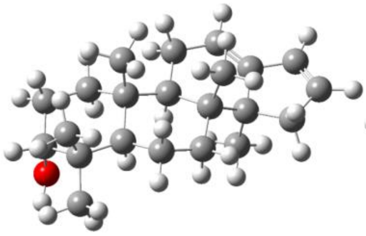

2-2 (55.13%)

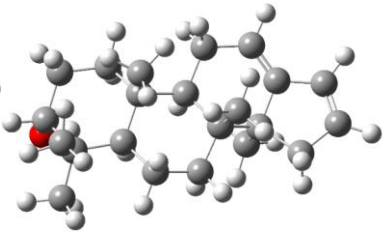

2-3 (15.35%)

Figure S48. The lowest energy conformers of **2** (the relative populations are in parentheses).

Selected conformation of 3 and their percentage

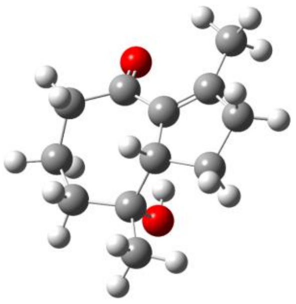

3-1 (18.18%)

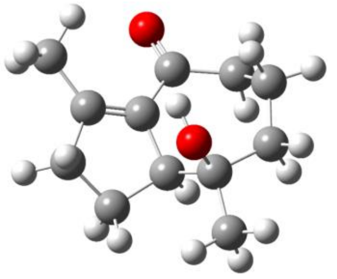

3-2 (74.34%)

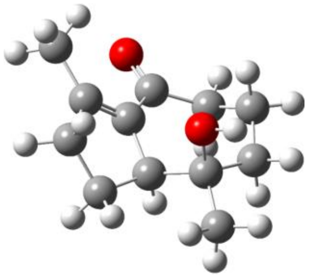

3-3 (6.67%)

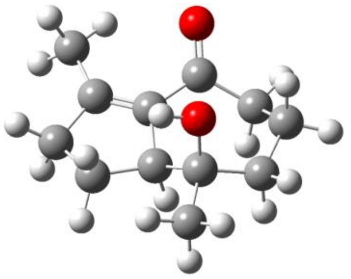

3-4 (0.81%)

Figure S49. The lowest energy conformers of **3** (the relative populations are in parentheses).

Selected conformation of 4 and their percentage

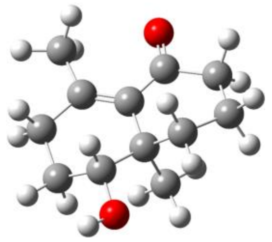

4-1 (16.75%)

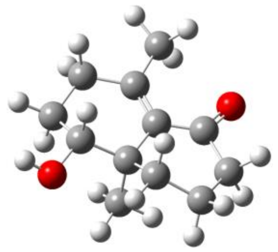

4-2 (41.45%)

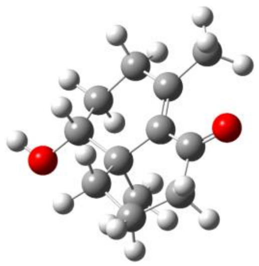

4-3 (41.80%)

Figure S50. The lowest energy conformers of **4** (the relative populations are in parentheses).

Table S1. Extracted heats and weighting factors of the optimized conformers of **1–4** at B3LYP/6-31+G(d) level

		B3LYP/6-31+G(d)	
	Conformer	Extracted heats	Boltzmann-calculated contribution (%)
**1**	1	-934.10609970	34.75%
	2	-934.10669391	65.25%
**2**	1	-934.09046291	29.52%
	2	-934.09105204	55.13%
	3	-934.08984497	15.35%
**3**	1	-618.61657993	18.18%
	2	-618.61790882	74.34%
	3	-618.61563400	6.67%
	4	-618.61363635	0.81%
**4**	1	-618.61160223	16.75%

	2	-618.61245730	41.45%
	3	-618.61246485	41.80%

Table S2. The Cartesian coordinates of the lowest energy conformers for **1–4**

**1-1**	X axis(Å)	Y axis(Å)	Z axis(Å)	**1-2**	X axis(Å)	Y axis(Å)	Z axis(Å)
C	1.2902	2.0117	0.3404	C	1.2942	2.0149	0.3221
C	0.5038	0.7197	-0.0285	C	0.5054	0.7211	-0.0354
C	1.3715	-0.5876	0.2343	C	1.3707	-0.5853	0.239
C	2.7259	-0.4698	-0.6043	C	2.7254	-0.4774	-0.6002
C	3.4014	0.8487	-0.2606	C	3.4034	0.8426	-0.268
C	2.6394	2.1178	-0.3817	C	2.6437	2.1121	-0.4007
C	3.8264	-1.4463	-0.2149	C	3.824	-1.4527	-0.202
C	4.9054	-0.7789	0.2226	C	4.9041	-0.7835	0.23
C	4.6432	0.6365	0.2002	C	4.6446	0.6322	0.1951
C	2.5556	-0.5734	-2.1369	C	2.5553	-0.5943	-2.1318
C	1.7189	-0.7591	1.7445	C	1.7172	-0.7443	1.7508
C	-0.9983	0.678	0.502	C	-0.9968	0.6869	0.4955
C	-1.7754	1.9017	-0.0774	C	-1.7708	1.907	-0.0945
C	-3.297	1.8251	0.092	C	-3.2928	1.834	0.0729
C	-3.8854	0.5396	-0.4839	C	-3.8851	0.5454	-0.4914
C	-3.2181	-0.7506	0.0766	C	-3.2203	-0.7413	0.0832
C	-1.6564	-0.632	-0.08	C	-1.658	-0.6267	-0.0749
C	-0.8678	-1.8902	0.3376	C	-0.8712	-1.8826	0.3535
C	0.5663	-1.84	-0.1985	C	0.5629	-1.8396	-0.1831
O	-3.7314	0.584	-1.9069	O	-3.728	0.6112	-1.9128
C	-1.064	0.7777	2.0453	C	-1.062	0.8006	2.0379
C	-3.6815	-0.9958	1.5259	C	-3.6836	-0.9649	1.5366
C	-3.7399	-1.9575	-0.7518	C	-3.7408	-1.9636	-0.7222
H	-1.5058	-0.5631	-1.1705	H	-1.5085	-0.5676	-1.1661
H	0.3843	0.7767	-1.1197	H	0.3861	0.7685	-1.1271
H	1.466	2.0672	1.4193	H	0.7169	2.9045	0.0515
H	0.7112	2.9026	0.0779	H	1.47	2.0798	1.4004
H	3.2088	2.9592	0.0294	H	3.2146	2.9561	0.0029
H	2.4699	2.3374	-1.4423	H	2.4748	2.3225	-1.4634
H	3.7469	-2.5204	-0.2966	H	3.7426	-2.5273	-0.2742
H	5.8324	-1.2173	0.5594	H	5.8303	-1.2204	0.571
H	5.3375	1.3868	0.5465	H	5.34	1.3844	0.5348
H	1.7888	0.102	-2.5255	H	1.7899	0.0792	-2.5267
H	3.4863	-0.3199	-2.6622	H	3.4866	-0.3473	-2.6592
H	2.2962	-1.5916	-2.4475	H	2.294	-1.6147	-2.4335
H	2.2554	0.1006	2.1551	H	2.2553	0.1178	2.154
H	0.8426	-0.9212	2.3688	H	0.8404	-0.8992	2.3762
H	2.3454	-1.6427	1.9109	H	2.3419	-1.6278	1.9251

H	-1.4345	2.8295	0.3974	H	-1.4293	2.838	0.3736
H	-1.5529	2.0046	-1.1476	H	-1.546	2.0016	-1.165
H	-3.7343	2.6895	-0.4239	H	-3.7276	2.6927	-0.455
H	-3.5752	1.9288	1.1449	H	-3.5727	1.9507	1.1241
H	-4.9641	0.5153	-0.286	H	-4.9638	0.5393	-0.2912
H	-0.8719	-2.0326	1.4212	H	-0.8757	-2.0157	1.4382
H	-1.3283	-2.7898	-0.0827	H	-1.3328	-2.7852	-0.0592
H	1.0928	-2.7539	0.1037	H	1.0878	-2.752	0.1267
H	0.49	-1.8826	-1.2903	H	0.4865	-1.8912	-1.2746
H	-4.2166	1.362	-2.2307	H	-4.3446	-0.0272	-2.309
H	-0.2239	1.3375	2.4626	H	-0.2194	1.3602	2.4504
H	-1.9517	1.3099	2.3962	H	-1.9472	1.3403	2.3838
H	-1.086	-0.1964	2.5358	H	-1.0883	-0.1691	2.537
H	-3.1721	-1.8559	1.9721	H	-3.1734	-1.8176	1.996
H	-4.7571	-1.21	1.5548	H	-4.7591	-1.1795	1.5683
H	-3.5287	-0.1369	2.1789	H	-3.5321	-0.0964	2.1768
H	-4.8329	-1.9296	-0.8371	H	-4.833	-1.9335	-0.8161
H	-3.3286	-1.9632	-1.7671	H	-3.3203	-1.9945	-1.7331
H	-3.485	-2.9141	-0.2836	H	-3.4937	-2.9107	-0.231
**2-1**	X axis(Å)	Y axis(Å)	Z axis(Å)	**2-2**	X axis(Å)	Y axis(Å)	Z axis(Å)
C	1.2388	2.0594	0.1874	C	1.2424	2.0618	0.1652
C	0.4778	0.7162	-0.044	C	0.4793	0.7173	-0.0524
C	1.3462	-0.5571	0.3316	C	1.3455	-0.5535	0.3359
C	2.7022	-0.4709	-0.5053	C	2.7021	-0.4777	-0.5009
C	3.3866	0.8539	-0.2025	C	3.3887	0.8484	-0.21
C	2.7389	1.9932	0.0654	C	2.7426	1.9914	0.0459
C	3.8118	-1.5096	-0.1718	C	3.8097	-1.5153	-0.1571
C	5.0653	-0.6808	-0.1126	C	5.0646	-0.6884	-0.1042
C	4.8026	0.63	-0.1028	C	4.8042	0.623	-0.1068
C	2.5036	-0.5256	-2.0528	C	2.5042	-0.5465	-2.0479
C	1.6598	-0.5951	1.857	C	1.6582	-0.5775	1.8618
C	-1.0305	0.7091	0.4583	C	-1.0289	0.7179	0.4503
C	-1.7866	1.8932	-0.2175	C	-1.7824	1.8969	-0.2363
C	-3.3109	1.8302	-0.0728	C	-3.307	1.837	-0.0928
C	-3.8896	0.5086	-0.5759	C	-3.8897	0.5127	-0.583
C	-3.2381	-0.7442	0.0821	C	-3.2401	-0.7353	0.0889
C	-1.6728	-0.6392	-0.0478	C	-1.6744	-0.6338	-0.0427
C	-0.8909	-1.864	0.4757	C	-0.894	-1.855	0.4919
C	0.562	-1.8471	-0.0162	C	0.559	-1.8452	0
O	-3.705	0.4587	-1.995	O	-3.7032	0.484	-2.002
C	-1.1306	0.9013	1.9925	C	-1.1279	0.9252	1.9826
C	-3.7434	-2.0028	-0.6761	C	-3.7445	-2.0081	-0.6446
C	-3.7353	-0.8916	1.5334	C	-3.7367	-0.8605	1.5431

H	-1.4977	-0.6476	-1.1367	H	-1.5006	-0.6529	-1.1317
H	0.3636	0.6675	-1.1354	H	0.3648	0.6577	-1.1432
H	0.8753	2.8055	-0.5297	H	0.8812	2.8006	-0.5606
H	1.0236	2.4602	1.1818	H	1.0266	2.4738	1.1548
H	3.2931	2.8954	0.31	H	3.2978	2.8953	0.282
H	3.9004	-2.2792	-0.947	H	3.8975	-2.292	-0.9253
H	3.6777	-2.0295	0.7777	H	3.6737	-2.0265	0.7969
H	6.0547	-1.1072	-0.0249	H	6.0532	-1.1155	-0.0116
H	5.5376	1.415	-0.0005	H	5.5404	1.4078	-0.0111
H	1.859	0.2696	-2.436	H	1.8615	0.2464	-2.4389
H	3.4594	-0.4119	-2.5819	H	3.4605	-0.4397	-2.5775
H	2.0925	-1.4863	-2.3761	H	2.0913	-1.5094	-2.3624
H	2.1457	0.317	2.2138	H	2.1445	0.3377	2.2104
H	0.769	-0.7416	2.4659	H	0.7671	-0.7179	2.4716
H	2.3073	-1.434	2.1235	H	2.3051	-1.4142	2.1366
H	-1.4404	2.8489	0.1957	H	-1.4355	2.8557	0.1689
H	-1.5458	1.9197	-1.2885	H	-1.54	1.9141	-1.3071
H	-3.7362	2.6589	-0.6531	H	-3.7302	2.6588	-0.6849
H	-3.6097	2.0023	0.9653	H	-3.6068	2.0225	0.9428
H	-4.9724	0.4985	-0.4003	H	-4.9724	0.5205	-0.4054
H	-0.9267	-1.9342	1.5657	H	-0.9301	-1.915	1.5825
H	-1.3381	-2.7912	0.1034	H	-1.3421	-2.7851	0.1281
H	1.0856	-2.7261	0.3801	H	1.0811	-2.7214	0.4045
H	0.5203	-1.9877	-1.1008	H	0.5172	-1.9956	-1.0832
H	-4.1778	1.2163	-2.38	H	-4.3121	-0.1793	-2.368
H	-0.2756	1.4395	2.407	H	-0.2706	1.4639	2.3918
H	-2.0018	1.492	2.2872	H	-1.9966	1.5227	2.2715
H	-1.2084	-0.0419	2.5359	H	-1.2095	-0.0123	2.5351
H	-3.4967	-2.9264	-0.1419	H	-3.5057	-2.9206	-0.0879
H	-4.8345	-1.9822	-0.784	H	-4.8346	-1.9861	-0.761
H	-3.3126	-2.0738	-1.6807	H	-3.3047	-2.1045	-1.6431
H	-3.5945	0.0092	2.1306	H	-3.5964	0.0492	2.1267
H	-4.8119	-1.1014	1.5518	H	-4.8133	-1.0704	1.5649
H	-3.2391	-1.721	2.0474	H	-3.2399	-1.6816	2.0697
**2-3**	X axis(Å)	Y axis(Å)	Z axis(Å)	**3-1**	X axis(Å)	Y axis(Å)	Z axis(Å)
C	1.2435	2.061	0.1775	C	2.4514	-1.9487	-0.0993
C	0.4814	0.7172	-0.048	C	1.5853	-0.6917	0.1238
C	1.3487	-0.5548	0.3354	C	0.192	-0.8872	-0.5437
C	2.7052	-0.4755	-0.5019	C	2.3804	0.5066	-0.4514
C	3.3894	0.8517	-0.2109	O	1.4704	-0.5525	1.5418
C	2.7427	1.993	0.0508	C	1.8271	1.9117	-0.2079
C	3.8151	-1.5111	-0.1597	C	0.4851	2.1581	-0.8749
C	5.0686	-0.6819	-0.1115	C	-0.6089	1.5534	-0.0401

C	4.8058	0.629	-0.1125	O	-1.1997	2.2049	0.8173
C	2.5065	-0.5435	-2.049	C	-0.89	0.1166	-0.2357
C	1.6622	-0.5832	1.8609	C	-2.1023	-0.4521	-0.1271
C	-1.0283	0.7143	0.4515	C	-0.5195	-2.1905	-0.1231
C	-1.7805	1.8957	-0.2325	C	-3.4305	0.1743	0.1261
C	-3.3051	1.8402	-0.0911	C	-2.0121	-1.9348	-0.3105
C	-3.9028	0.514	-0.5585	H	0.3307	-0.9066	-1.6344
C	-3.2364	-0.7413	0.0826	H	2.5628	-2.1772	-1.1644
C	-1.6709	-0.6363	-0.0467	H	3.4514	-1.8155	0.3308
C	-0.8897	-1.8581	0.4866	H	2.0318	-2.8249	0.4066
C	0.5634	-1.8461	-0.0047	H	2.5218	0.3638	-1.531
O	-3.8329	0.4685	-1.9873	H	3.385	0.4995	-0.006
C	-1.1315	0.9161	1.9843	H	0.8199	0.1397	1.7548
C	-3.7423	-1.9974	-0.6781	H	2.549	2.6291	-0.6179
C	-3.7256	-0.8953	1.5371	H	1.7705	2.1098	0.8693
H	-1.4915	-0.6552	-1.1338	H	0.2895	3.2347	-0.945
H	0.3689	0.6629	-1.1393	H	0.4492	1.7661	-1.897
H	0.8785	2.8052	-0.5408	H	-0.1875	-3.0496	-0.7161
H	1.0317	2.4646	1.1714	H	-0.3239	-2.4243	0.932
H	3.2983	2.8965	0.2881	H	-3.8071	-0.1213	1.1102
H	3.9019	-2.2889	-0.927	H	-3.4002	1.2659	0.0853
H	3.683	-2.0207	0.7957	H	-4.1503	-0.1549	-0.6307
H	6.0584	-1.1072	-0.022	H	-2.5958	-2.4848	0.4339
H	5.5412	1.4148	-0.0184	H	-2.349	-2.2188	-1.3131
H	1.8597	0.2468	-2.4386				
H	3.4618	-0.4318	-2.5793				
H	2.0978	-1.508	-2.3642				
H	2.1536	0.3287	2.2109				
H	0.771	-0.7199	2.4712				
H	2.3048	-1.424	2.1333				
H	-1.4332	2.8523	0.1779				
H	-1.5339	1.9203	-1.3018				
H	-3.7278	2.6585	-0.688				
H	-3.5988	2.0353	0.9449				
H	-4.9736	0.5176	-0.3207				
H	-0.925	-1.9201	1.5771				
H	-1.3376	-2.7877	0.1213				
H	1.0859	-2.723	0.3976				
H	0.522	-1.9937	-1.0882				
H	-2.9004	0.48	-2.2565				
H	-0.2816	1.4646	2.3958				
H	-2.0083	1.5009	2.2741				
H	-1.202	-0.0236	2.5345				

H	-3.4891	-2.923	-0.1508				
H	-4.8341	-1.9801	-0.7788				
H	-3.3175	-2.0607	-1.6858				
H	-3.5896	0.0074	2.1329				
H	-4.8006	-1.1138	1.5606				
H	-3.2216	-1.7212	2.0487				
**3-2**	X axis(Å)	Y axis(Å)	Z axis(Å)	**3-3**	X axis(Å)	Y axis(Å)	Z axis(Å)
C	3.0304	-0.6223	-0.6757	C	3.0288	-0.5853	-0.7243
C	1.7425	-0.1182	0.0052	C	1.7451	-0.0961	-0.0255
C	0.4964	-0.5248	-0.8339	C	0.4905	-0.536	-0.8331
C	1.9016	1.4068	0.1971	C	1.8609	1.4347	0.145
O	1.7194	-0.7404	1.2952	O	1.7188	-0.6989	1.2734
C	0.7284	2.1459	0.8434	C	0.7043	2.1107	0.8844
C	-0.4028	2.4404	-0.1334	C	-0.4752	2.4328	-0.0235
C	-1.3346	1.2835	-0.3995	C	-1.3491	1.2632	-0.4068
O	-2.4895	1.4806	-0.776	O	-2.4704	1.4494	-0.8808
C	-0.826	-0.0965	-0.2556	C	-0.8315	-0.1095	-0.2548
C	-1.517	-1.1109	0.289	C	-1.5187	-1.1183	0.3037
C	0.2693	-2.0508	-0.9231	C	0.2815	-2.0668	-0.8823
C	-2.8476	-1.0997	0.959	C	-2.8471	-1.0999	0.9757
C	-0.7742	-2.4006	0.1371	C	-0.7595	-2.4008	0.1862
H	0.6041	-0.122	-1.8495	H	0.58	-0.1578	-1.8601
H	3.1149	-0.25	-1.702	H	3.0875	-0.2221	-1.7556
H	3.9171	-0.2952	-0.1191	H	3.9201	-0.2368	-0.1887
H	3.0868	-1.7147	-0.6938	H	3.1021	-1.6768	-0.7319
H	2.1409	1.8807	-0.7637	H	2.0105	1.9134	-0.8312
H	2.769	1.5751	0.8508	H	2.7656	1.6428	0.7334
H	0.8802	-0.5206	1.7346	H	2.5229	-0.433	1.7522
H	1.1098	3.1118	1.1999	H	1.0789	3.0638	1.2799
H	0.3628	1.6154	1.7293	H	0.3888	1.5202	1.7521
H	-1.0182	3.2385	0.3008	H	-1.1233	3.1404	0.5087
H	-0.0179	2.8036	-1.0924	H	-0.1355	2.9206	-0.944
H	-0.1357	-2.2921	-1.9163	H	-0.1186	-2.3395	-1.8692
H	1.1708	-2.656	-0.8029	H	1.1905	-2.657	-0.744
H	-2.7758	-1.5754	1.9426	H	-2.7672	-1.5336	1.9778
H	-3.2316	-0.0884	1.1172	H	-3.2467	-0.0887	1.0905
H	-3.5782	-1.6532	0.3611	H	-3.5702	-1.6882	0.4022
H	-0.3106	-2.6932	1.0848	H	-0.2931	-2.6621	1.1417
H	-1.419	-3.2136	-0.2105	H	-1.3932	-3.2311	-0.1402
**3-4**	X axis(Å)	Y axis(Å)	Z axis(Å)	**4-1**	X axis(Å)	Y axis(Å)	Z axis(Å)
C	2.4379	-1.9672	-0.0124	C	-1.926	-0.2466	-0.3827
C	1.5853	-0.6929	0.1285	C	-0.53	-0.7035	0.1437
C	0.1933	-0.8842	-0.542	C	-2.31	1.1323	0.1459

C	2.3841	0.4736	-0.5028	C	0.156	1.7236	-0.1933
O	1.4601	-0.4216	1.5254	C	0.4827	0.4291	0.0203
C	1.8585	1.8878	-0.2408	C	2.3353	-1.2026	-0.6466
C	0.4963	2.1566	-0.854	C	-0.0788	-1.922	-0.7112
C	-0.5994	1.5551	-0.0129	C	1.3636	-2.3551	-0.4493
O	-1.2353	2.2295	0.7938	C	-0.6203	-1.1232	1.6319
C	-0.8838	0.1155	-0.2083	O	-2.9356	-1.1907	-0.0265
C	-2.0977	-0.4542	-0.1155	C	1.9158	0.0043	0.1565
C	-0.5218	-2.199	-0.1639	C	-1.2844	2.1803	-0.2726
C	-3.4273	0.1695	0.1393	C	1.1492	2.8424	-0.3899
C	-2.0137	-1.9313	-0.3397	O	2.7071	0.6245	0.8617
H	0.3325	-0.8812	-1.633	H	-1.9096	-0.2021	-1.48
H	2.5584	-2.2558	-1.0616	H	-2.4089	1.1103	1.2378
H	3.434	-1.8203	0.4219	H	-3.3029	1.4175	-0.2237
H	2.0025	-2.8088	0.5367	H	3.3382	-1.5092	-0.33
H	2.48	0.3145	-1.5847	H	2.3928	-0.9095	-1.7009
H	3.4057	0.4548	-0.0983	H	-0.7439	-2.7766	-0.5335
H	0.8895	-1.0928	1.9356	H	-0.1695	-1.6776	-1.7786
H	2.5753	2.5945	-0.6778	H	1.4565	-2.7512	0.5683
H	1.8429	2.089	0.8371	H	1.6277	-3.1746	-1.1277
H	0.3168	3.2377	-0.8997	H	-1.2865	-1.9826	1.7659
H	0.427	1.7884	-1.8835	H	0.3551	-1.4066	2.0425
H	-0.1928	-3.0371	-0.788	H	-0.9968	-0.3097	2.2611
H	-0.3308	-2.4763	0.8803	H	-3.7835	-0.8649	-0.3747
H	-3.8073	-0.1375	1.1186	H	-1.4252	3.0706	0.3525
H	-3.3999	1.2612	0.1099	H	-1.4893	2.4821	-1.3078
H	-4.1442	-0.1524	-0.6234	H	0.8305	3.4867	-1.2171
H	-2.5981	-2.4989	0.3908	H	1.2118	3.4538	0.5157
H	-2.3543	-2.1867	-1.3487	H	2.1523	2.494	-0.6475
**4-2**	X axis(Å)	Y axis(Å)	Z axis(Å)	**4-3**	X axis(Å)	Y axis(Å)	Z axis(Å)
C	-1.9271	-0.2502	-0.3908	C	-1.9355	-0.2263	-0.3808
C	-0.5302	-0.7067	0.1349	C	-0.541	-0.6979	0.1417
C	-2.3155	1.1307	0.13	C	-2.2983	1.1596	0.1429
C	0.1499	1.7248	-0.1934	C	0.1707	1.723	-0.1978
C	0.4778	0.4312	0.0228	C	0.4825	0.4263	0.0225
C	2.341	-1.1841	-0.6489	C	2.3261	-1.2103	-0.6482
C	-0.0705	-1.9151	-0.7298	C	-0.0942	-1.9148	-0.7187
C	1.3737	-2.3435	-0.4675	C	1.3465	-2.3574	-0.4599
C	-0.618	-1.1398	1.6191	C	-0.6361	-1.1246	1.6271
O	-2.9499	-1.1914	-0.0649	O	-2.9805	-1.1236	-0.0059
C	1.9108	0.01	0.1674	C	1.9114	-0.0091	0.1651
C	-1.2908	2.1779	-0.2917	C	-1.2647	2.1936	-0.2868
C	1.1424	2.8459	-0.381	C	1.1754	2.8319	-0.3922

O	2.6894	0.6191	0.8955	O	2.7014	0.5954	0.8845
H	-1.9034	-0.199	-1.4874	H	-1.9324	-0.1807	-1.4783
H	-2.4131	1.1159	1.2223	H	-2.3899	1.1451	1.2358
H	-3.3093	1.4088	-0.2424	H	-3.291	1.4526	-0.2214
H	3.3445	-1.4887	-0.3324	H	3.3269	-1.5252	-0.3335
H	2.4004	-0.8784	-1.6994	H	2.3858	-0.9088	-1.6999
H	-0.7322	-2.7742	-0.5621	H	-0.7595	-2.7693	-0.5439
H	-0.1589	-1.6613	-1.7952	H	-0.1854	-1.6653	-1.7848
H	1.4647	-2.7496	0.5464	H	1.4364	-2.7612	0.5551
H	1.6443	-3.1546	-1.1534	H	1.6058	-3.1739	-1.1437
H	-1.2625	-2.0171	1.7426	H	-1.3102	-1.9787	1.7552
H	0.3615	-1.4029	2.0336	H	0.3362	-1.4189	2.0372
H	-1.0187	-0.3404	2.2515	H	-1.0065	-0.3111	2.26
H	-3.1969	-1.0666	0.8677	H	-2.8602	-1.9508	-0.5019
H	-1.4397	3.0741	0.323	H	-1.3984	3.0922	0.3279
H	-1.487	2.4684	-1.3319	H	-1.4644	2.4854	-1.3259
H	2.152	2.5	-0.6153	H	2.1788	2.4734	-0.6342
H	0.8368	3.4816	-1.2197	H	0.8717	3.4715	-1.2285
H	1.1851	3.4653	0.5204	H	1.2323	3.4505	0.509

Reference

(1) M. J. Frisch, G. W. Trucks, H. B. Schlegel, G. E. Scuseria, M. A. Robb, J. R. Cheeseman, G. Scalmani, V. Barone, B. Mennucci, G. A. Petersson, H. Nakatsuji, M. Caricato, X. Li, H. P. Hratchian, A. F. Izmaylov, J. Bloino, G. Zheng, J. L. Sonnenberg, M. Hada, M. Ehara, K. Toyota, R. Fukuda, J. Hasegawa, M. Ishida, T. Nakajima, Y. Honda, O. Kitao, H. Nakai, T. Vreven, J. A. Montgomery, J. E. Peralta, F. Ogliaro, M. Bearpark, J. J. Heyd, E. Brothers, K. N. Kudin, V. N. Staroverov, R. Kobayashi, J. Normand, K. Raghavachari, A. Rendell, J. C. Burant, S. S. Iyengar, J. Tomasi, M. Cossi, N. Rega, J. M. Millam, M. Klene, J. E. Knox, J. B. Cross, V. Bakken, C. Adamo, J. Jaramillo, R. Gomperts, R. E. Stratmann, O. Yazyev, A. J. Austin, R. Cammi, C. Pomelli, J. W. Ochterski, R. L. Martin, K. Morokuma, V. G. Zakrzewski, G. A. Voth, P. Salvador, J. J. Dannenberg, S. Dapprich, A. D. Daniels, O. Farkas, J. B. Foresman, J. V. Ortiz, J. Cioslowski, D. J. Fox, Gaussian, Inc., Wallingford CT, 2009.
